# Supplementary figures and images for: Short-chain fatty acids bind to apoptosis-associated speck-like protein to activate inflammasome complex to prevent Salmonella infection
Source: PLoS Biol. 2020 Sep 29;18(9):e3000813. doi: 10.1371/journal.pbio.3000813 (PMC7524008; doi:10.1371/journal.pbio.3000813)

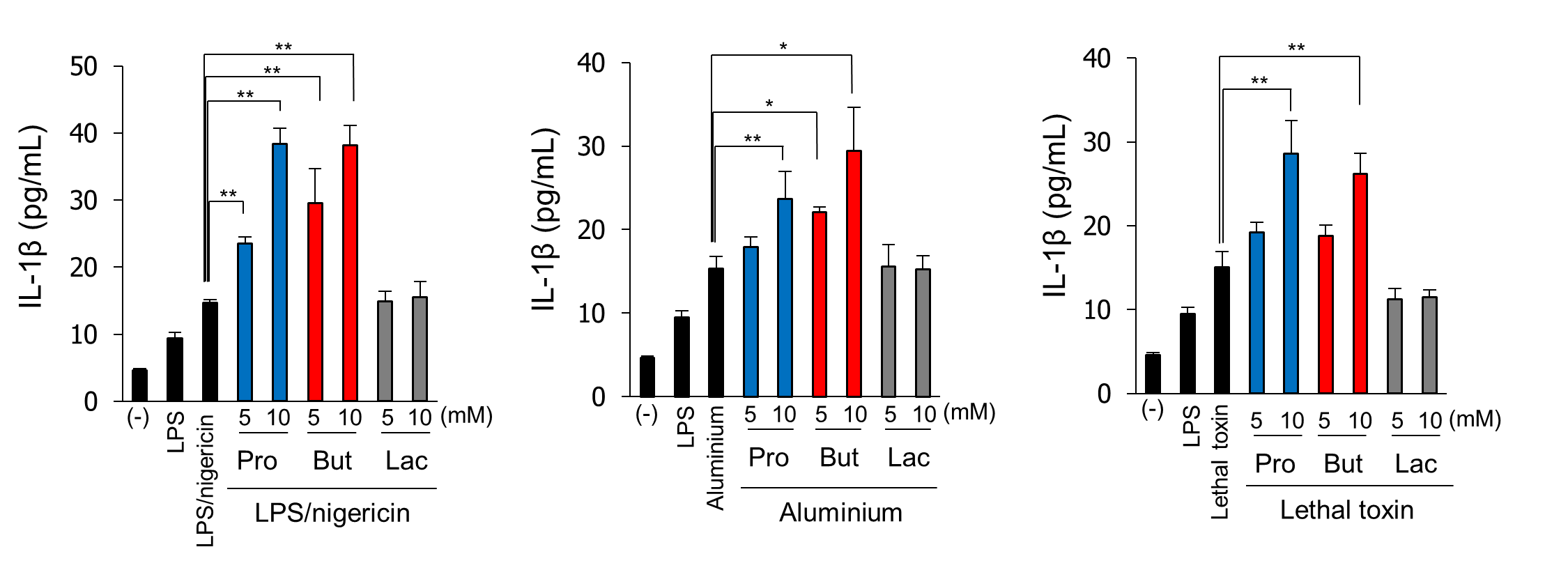

Supplement: S1 Fig — IL-1β production in U937 cells stimulated with SCFAs and LPS/nigericin, alum adjuvant, or lethal toxin as measured by ELISA. Data are the mean ± SD of three independent assays. One-way ANOVA analysis, *P < 0.05, **P < 0.01. Data are listed in S1 Data. ANOVA, analysis of variance; IL, interleukin; LPS, lipopolysaccharide; NLRP, nucleotide-binding oligomerization domain-like receptor protein; SCFA, short-chain fatty acid; SD, standard deviation. (TIF) [file pbio.3000813.s001.tif]

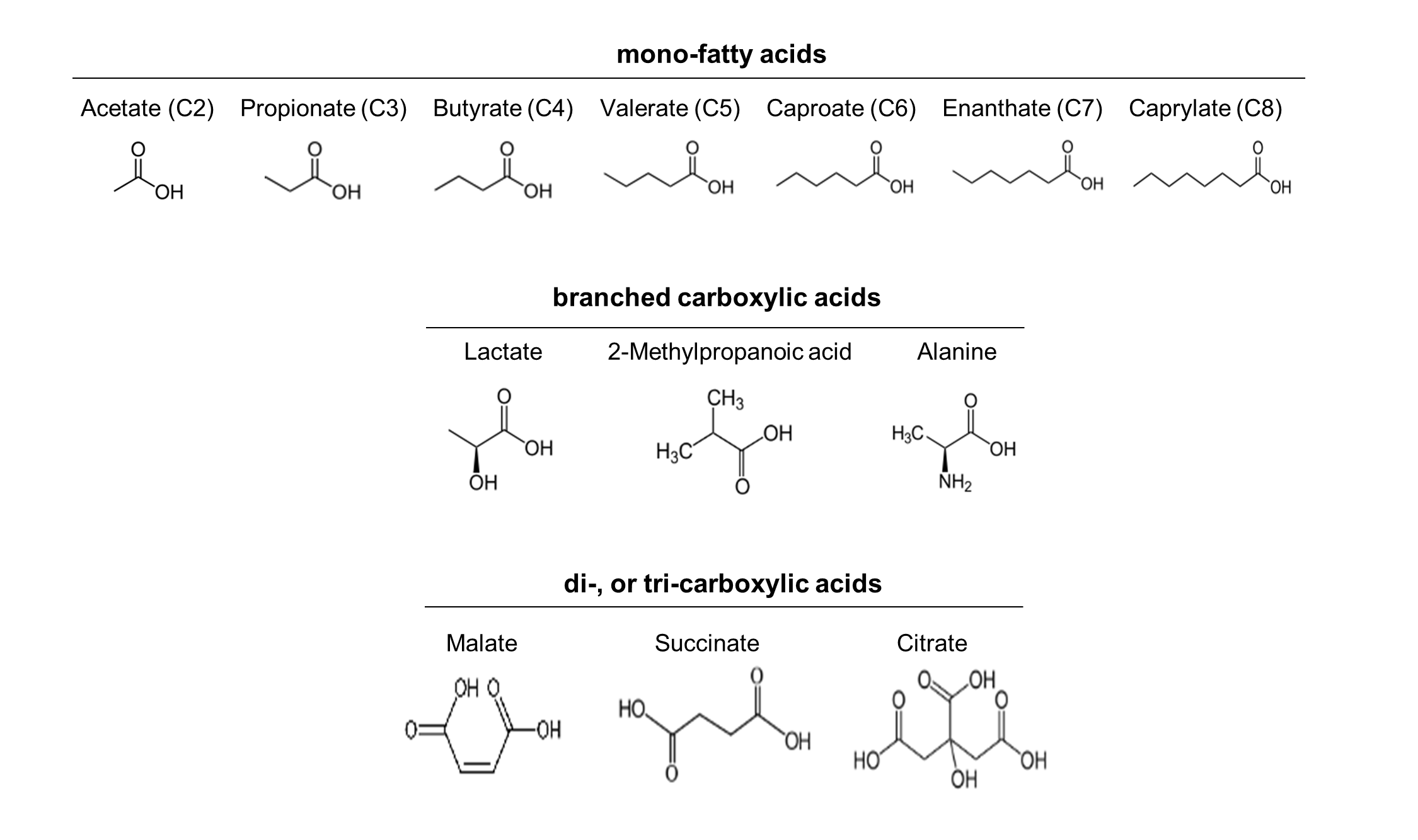

Supplement: S2 Fig — (TIF) [file pbio.3000813.s002.tif]

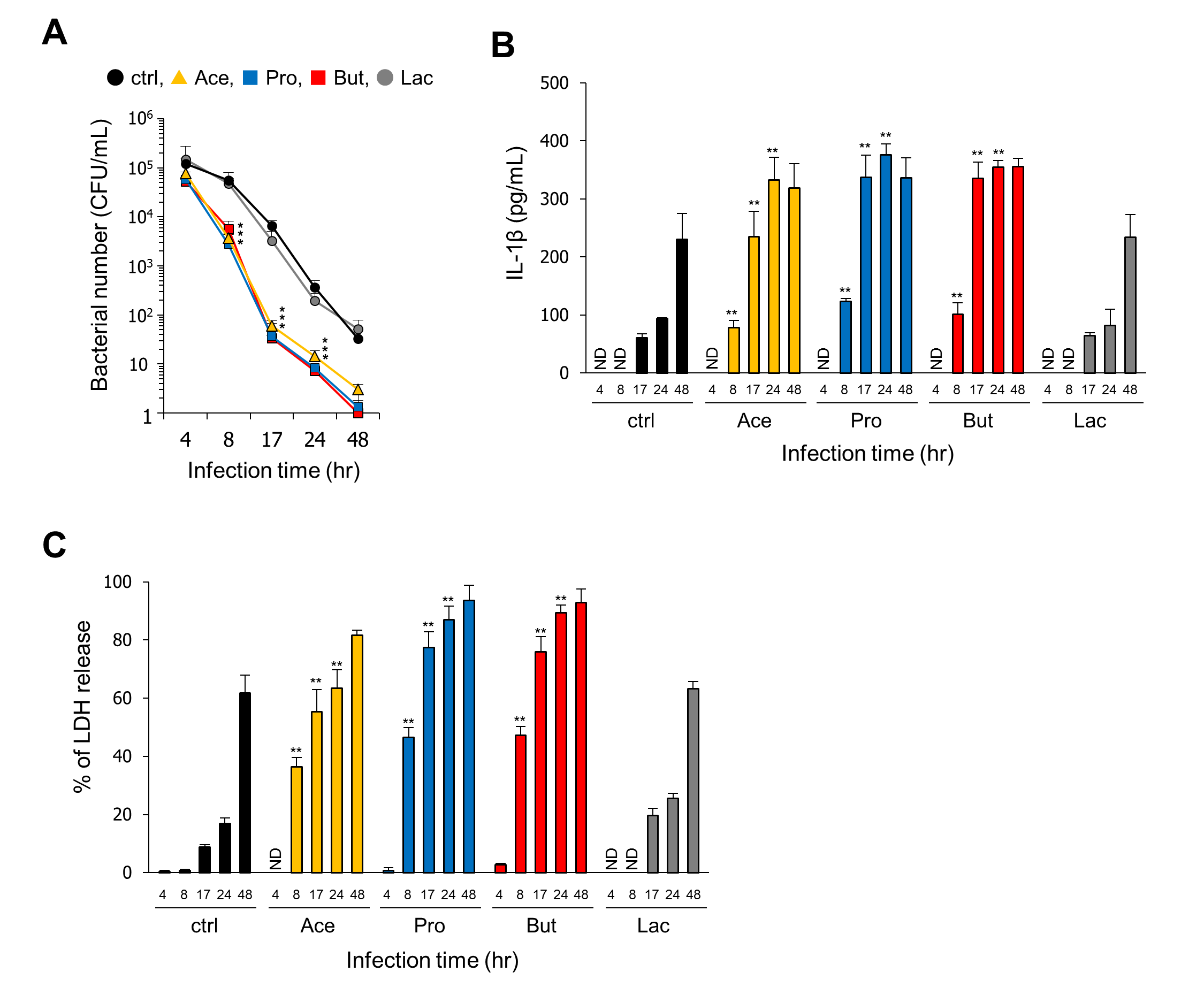

Supplement: S3 Fig — (A) BMDMs derived from wild-type mice were infected with S. Typhimurium strain A at multiplicity of infection of 5 for 10 minutes and then incubated in DMEM containing 100 μg/mL gentamycin for the indicated time with or without treatment with 10 mM acetate (Ace), propionate (Pro), butyrate (But), or lactate (Lac). Bacterial cell numbers within BMDMs are shown. Data are the mean ± SD of three independent assays. One-way ANOVA analysis, *P < 0.05 versus nontreated BMDMs (ctrl). Data are listed in S1 Data. (B and C) IL-1β production and LDH release in cell-culture media as determined by LDH assay and ELISA, respectively. Data are the mean ± SD of three independent assays. One-way ANOVA analysis, **P < 0.01 versus nontreated BMDMs (ctrl). Data listed in S1 Data. ANOVA, analysis of variance; BMDM, bone marrow–derived macrophage; DMEM, Dulbecco’s modified Eagle’s medium; IL, interleukin; LDH, lactate dehydrogenase; ND, not detected (below the detection limit); S. Typhimurium, S. enterica serovar Typhimurium; SCFA, short-chain fatty acid; SD, standard deviation. (TIF) [file pbio.3000813.s003.tif]

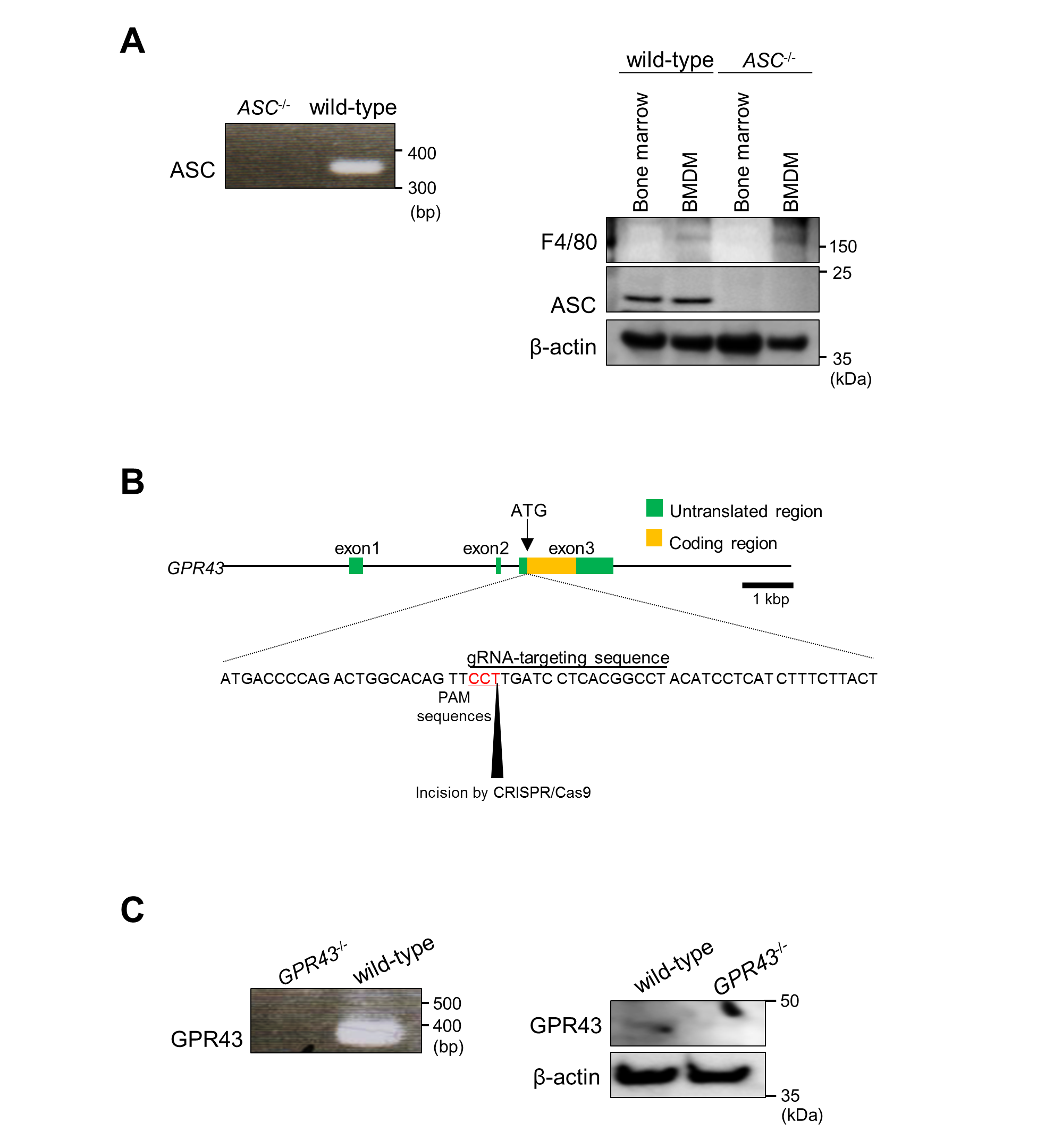

Supplement: S4 Fig — (A) PCR analysis of ASC (350-bp PCR product) in genomic DNA isolated from wild-type or ASC–/–mice (left panel). The expression of ASC and F4/80, a macrophage marker, in the bone marrow or in BMDMs derived from wild-type or ASC–/–mice was analyzed by western blotting (right panel). (B) GPR43-deficient mice were generated using CRISPR-Cas9 gene editing. Cas9/gRNA-targeting sites in GPR43. Exons are indicated by closed boxes, and the boxed sequence begins 60 bp from the start codon and contains the targeting sequence in the coding region of exon 3. The gRNA-targeting sequence is underlined, and the PAM sequences are indicated in red. The CRISPR-Cas9 incision site is indicated by an arrow. (C) PCR analysis of GPR43 (390-bp PCR product) in genomic DNA isolated from wild-type or GPR43–/–mice (left panel). GPR43 expression in BMDMs derived from wild-type or GPR43–/–mice was analyzed by western blotting (right panel). ASC, apoptosis-associated speck-like protein; BMDM, bone marrow–derived macrophage; CRISPR-Cas9, clustered regularly interspersed short palindromic repeats–CRISPR-associated protein 9; gRNA, guide RNA; PAM, protospacer adjacent motif; PCR, polymerase chain reaction. (TIF) [file pbio.3000813.s004.tif]

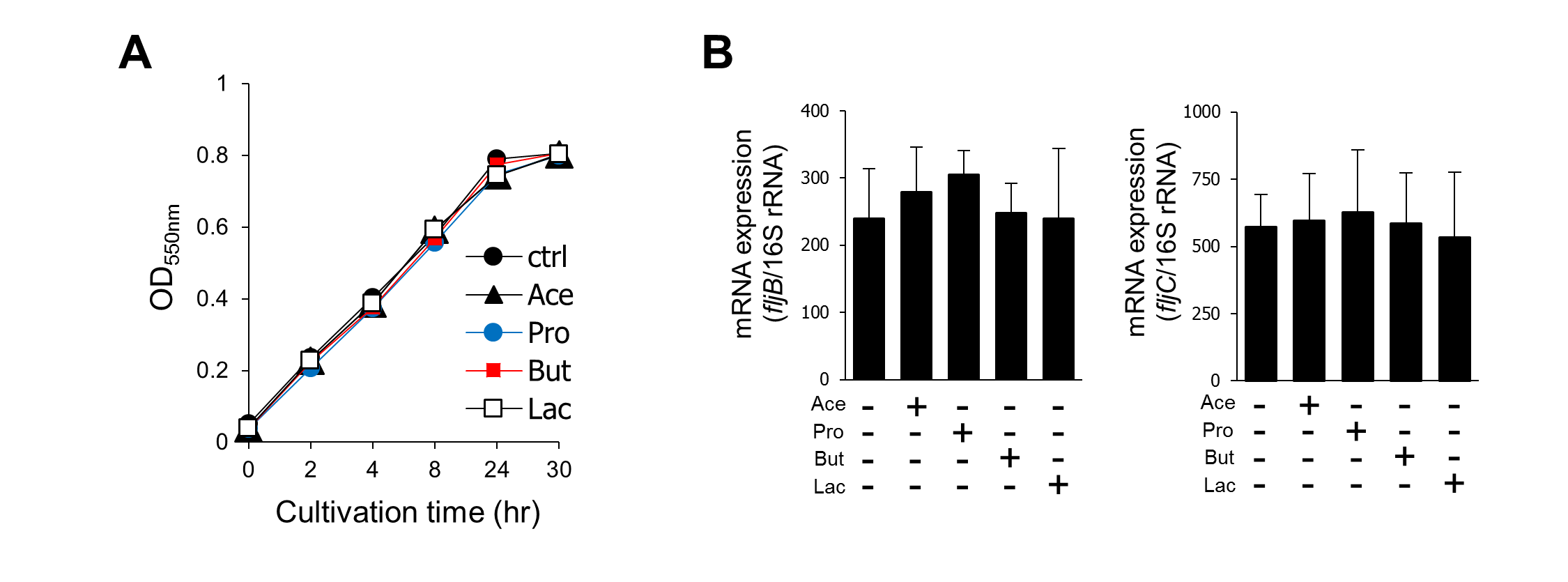

Supplement: S5 Fig — (A) S. Typhimurium strain A was cultured in LB broth containing 10 mM SCFAs (acetate, propionate, butyrate, or lactate) for the indicated times at 37°C, with agitation. Bacterial growth was monitored by measuring the optical density at 550 nm. Data are listed in S1 Data. (B) Effects of SCFAs on the expression of two flagellin genes of S. Typhimurium strain A. mRNA expression of fliC and fliB was measured by RT-qPCR. Data are the mean ± SD of three independent assays. Data are listed in S1 Data. LB, Luria–Bertani; RT-qPCR, quantitative reverse transcription–polymerase chain reaction; S. Typhimurium, S. enterica serovar Typhimurium; SCFA, short-chain fatty acid; SD, standard deviation. (TIF) [file pbio.3000813.s005.tif]

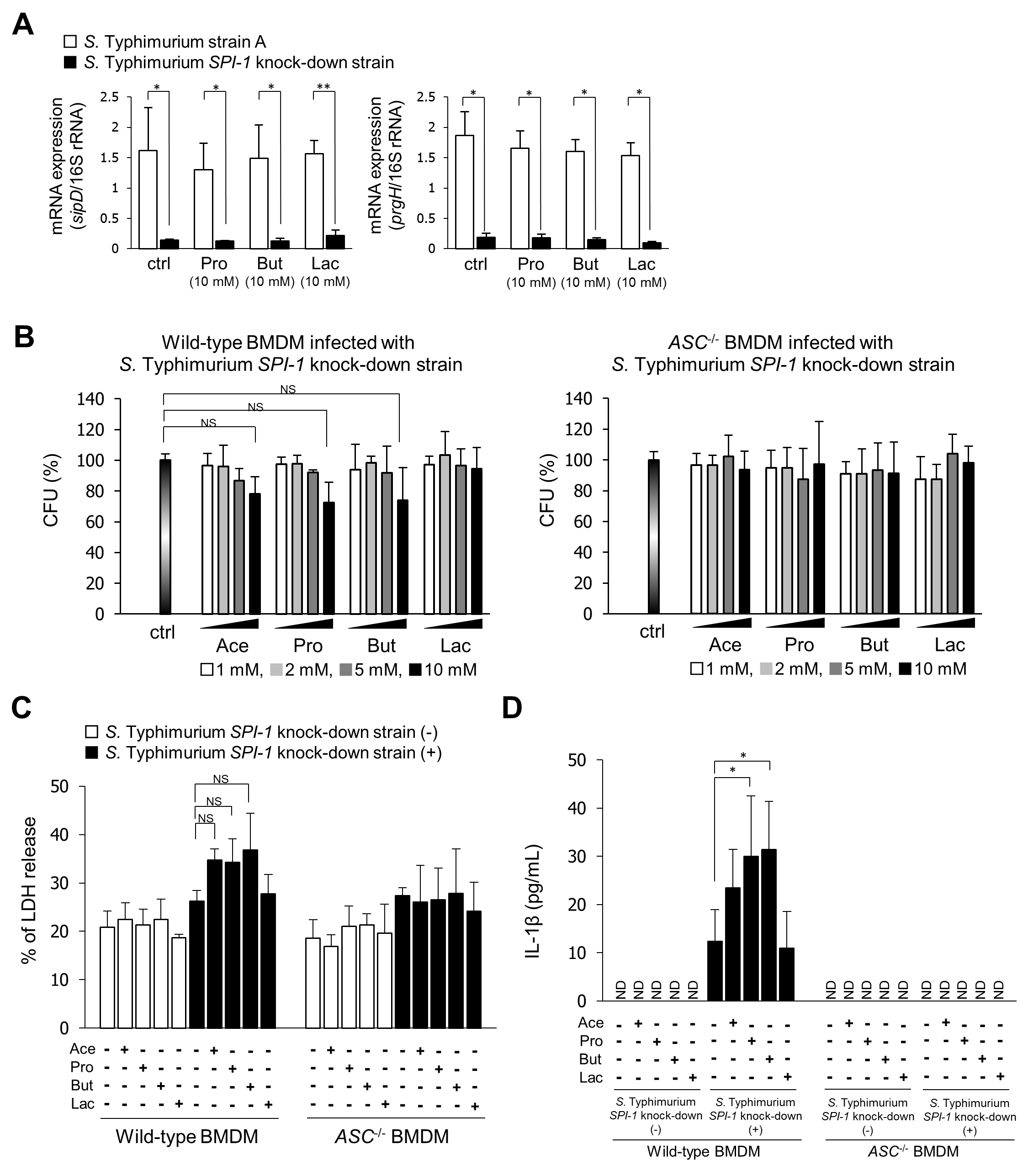

Supplement: S6 Fig — (A) Effects of SCFAs on the expression of SPI-1 genes (sipD and prgH) by S. Typhimurium. The expression of sipD and prgH in S. Typhimurium strain A and the S. Typhimurium SPI-1 knock-down strain were measured by RT-qPCR after SCFA exposure. Data are the mean ± SD of three independent assays. Student's t test, *P < 0.05, **P < 0.01. Data are listed in S1 Data. (B) BMDMs derived from wild-type or ASC−/− mice were infected with SPI-1 knock-down S. Typhimurium at a multiplicity of infection of 5 for 10 minutes and then were incubated in DMEM containing 100 μg/mL gentamycin for 15 hours with or without treatment with acetate (Ace), propionate (Pro), butyrate (But), or lactate (Lac). The percentages of surviving S. Typhimurium SPI-1 knock-down bacteria in SCFA-treated macrophages is shown relative to the survival in untreated macrophages (ctrl). Data are the mean ± SD of three independent assays. Data are listed in S1 Data. (C and D) BMDMs derived from wild-type or ASC−/− mice were infected with SPI-1 knock-down S. Typhimurium at a multiplicity of infection of 5 for 10 minutes and then were incubated in DMEM containing 100 μg/mL gentamycin for 15 hours with or without treatment with 10 mM acetate (Ace), 10 mM propionate (Pro), 10 mM butyrate (But), or 10 mM lactate (Lac). Cell supernatants were collected, and LDH release and IL-1β production were determined by LDH assay and ELISA, respectively. Data are the mean ± SD of three independent assays. One-way ANOVA analysis, *P < 0.05. Data are listed in S1 Data. ANOVA, analysis of variance; BMDM, bone marrow–derived macrophage; DMEM, Dulbecco’s modified Eagle’s medium; LDH, lactate dehydrogenase; ND, not detected (below the detection limit); NS, not significant; RT-qPCR, quantitative reverse transcription–polymerase chain reaction; S. Typhimurium, S. enterica serovar Typhimurium; SCFA, short-chain fatty acid; SD, standard deviation; SPI-1, Salmonella pathogenicity island 1. (TIF) [file pbio.3000813.s006.tif]

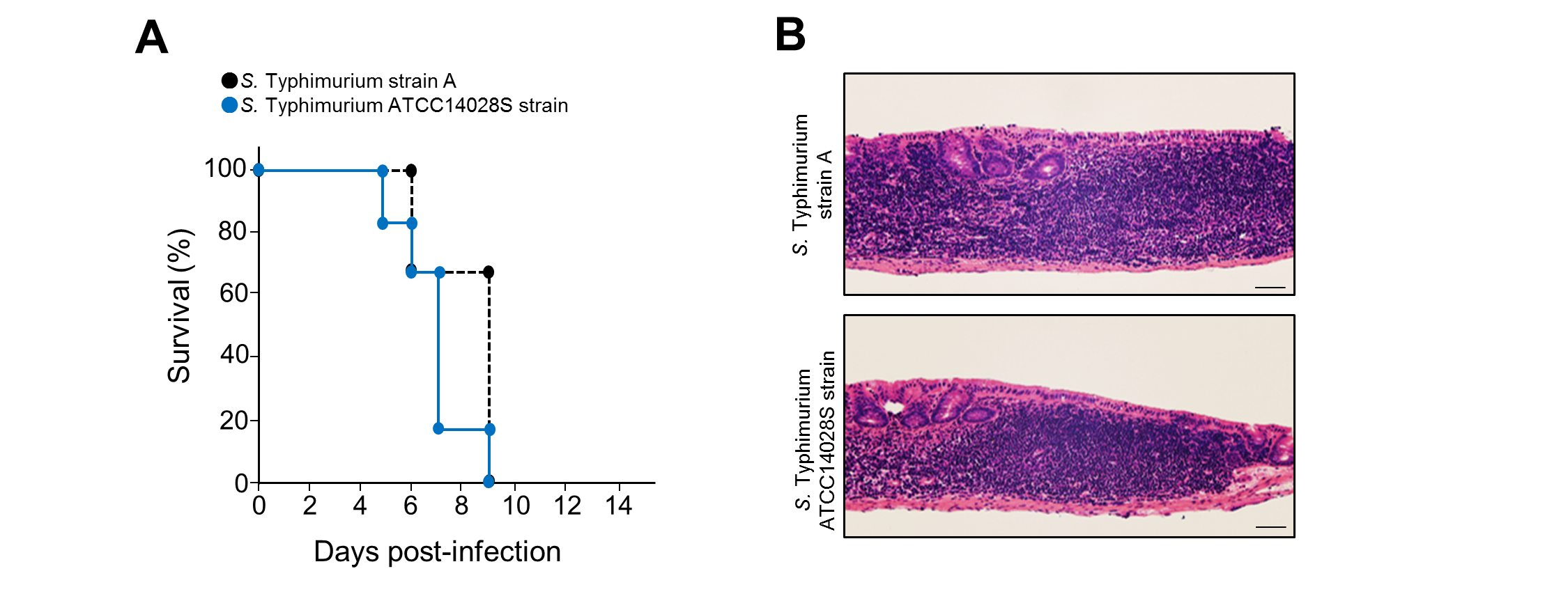

Supplement: S7 Fig — (A) Survival of SPF wild-type mice infected with S. Typhimurium strain A (108 bacteria) (black circle) and infected with S. Typhimurium ATCC14028S strain (108 bacteria) (blue circle). n = 6 per group. Data listed in S1 Data. (B) HE staining of cecum tissues from SPF wild-type infected with S. Typhimurium strain A or S. Typhimurium ATCC14028S strain. Scale bars = 50 μm. HE, hematoxylin–eosin; S. Typhimurium, S. enterica serovar Typhimurium; SPF, specific pathogen-free. (TIF) [file pbio.3000813.s007.tif]

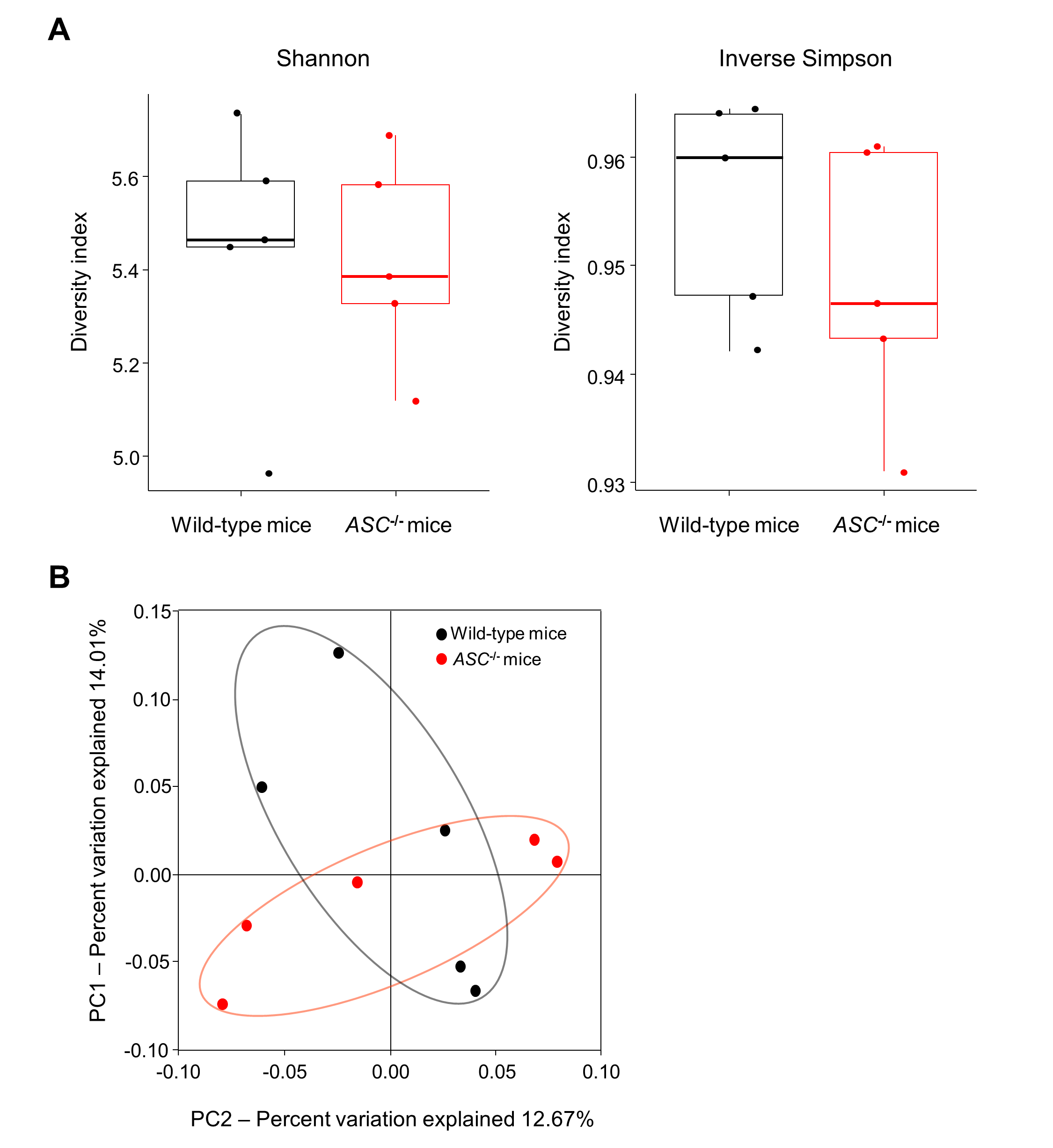

Supplement: S8 Fig — (A) The Shannon diversity index and the Inverse Simpson index were analyzed to compare the microbial diversity between wild-type and ASC−/− mice. The box-and-whisker plots show the full range of variation, the interquartile ranges, and the median values. The data points indicate the diversity indices of each mouse. Data are listed in S1 Data. (B) The two principal coordinates from the PCoA of the weighted UniFrac distances were plotted to the distance matrices for visualization of the microbial diversity. The data points indicate each mouse. Data are listed in S1 Data. ASC, apoptosis-associated speck-like protein; PCoA, principal coordinate analysis. (TIF) [file pbio.3000813.s008.tif]

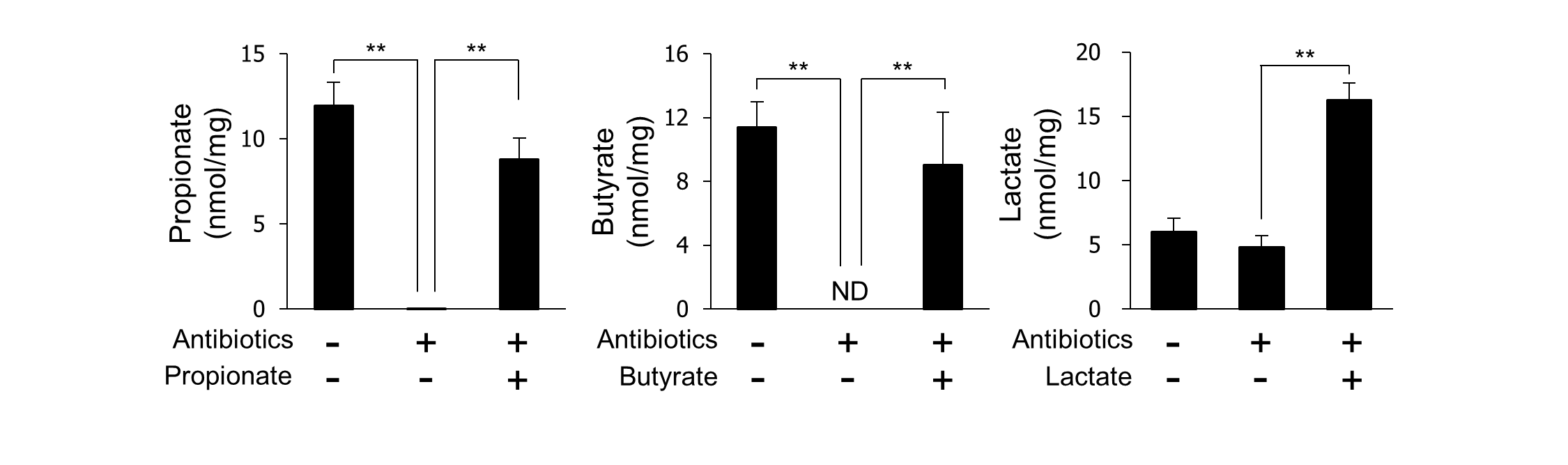

Supplement: S9 Fig — Mice were given drinking water containing ampicillin (1 g/L), metronidazole (1 g/L), neomycin (1 g/L), and vancomycin (0.5 g/L). After 4 weeks, the mice were humanely killed and cecum samples were collected. Antibiotic-treated mice were given drinking water containing 300 mM propionate, butyrate, or lactate for 1 week, and cecum samples were collected following a boost with propionate, butyrate, and lactate 2 hours prior to killing. n = 5 per group. One-way ANOVA analysis, **P < 0.01. Data are listed in S1 Data. ND, not detected (below the detection limit). (TIF) [file pbio.3000813.s009.tif]

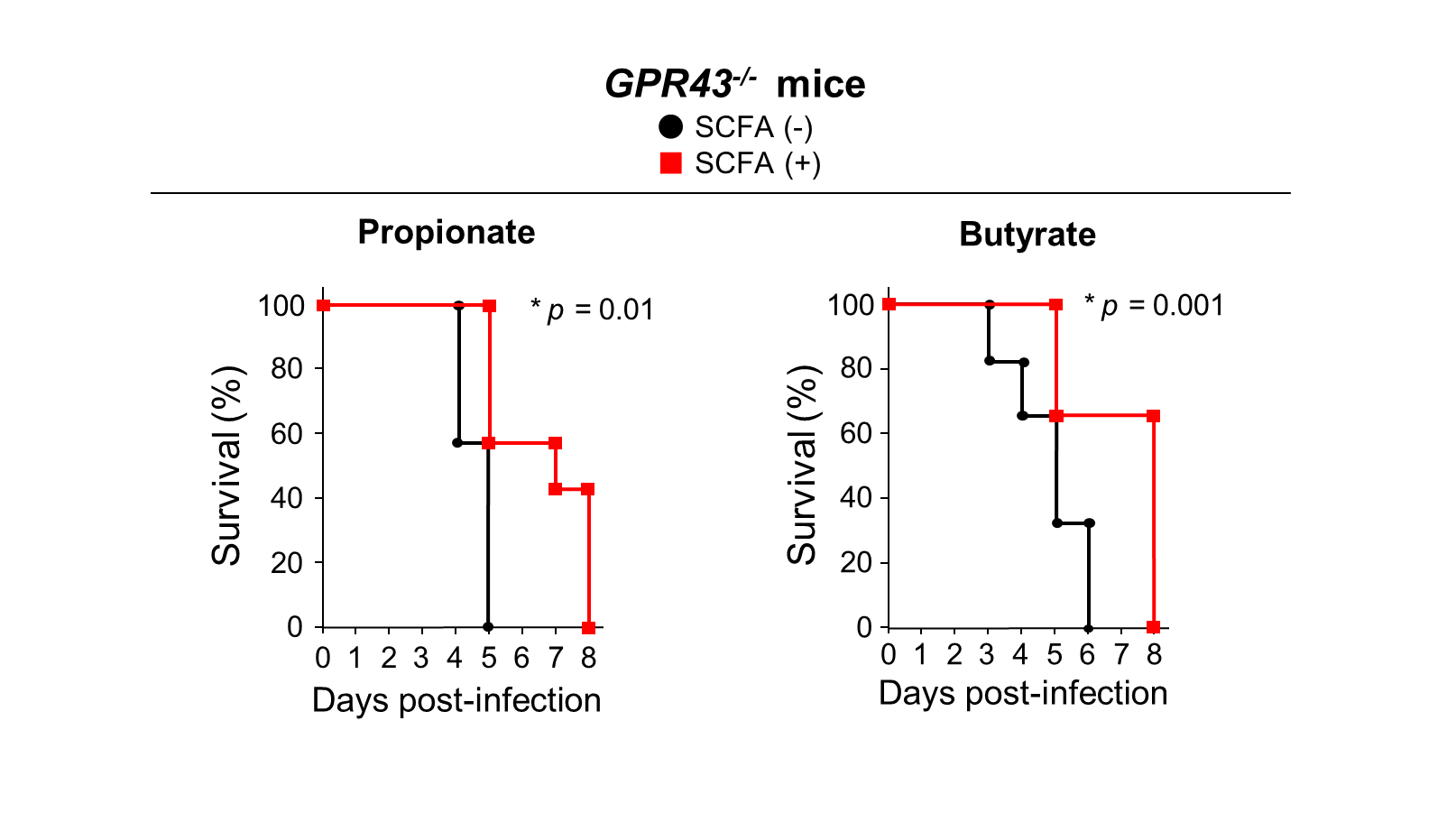

Supplement: S10 Fig — GRP43–/–mice treated or not with propionate (n = 7 mice per group) or butyrate (n = 6 mice per group) were infected orally with 108 S. Typhimurium, and survival was monitored. P-values were determined by the log-rank test. Data are listed in S1 Data. S. Typhimurium, S. enterica serovar Typhimurium. (TIF) [file pbio.3000813.s010.tif]

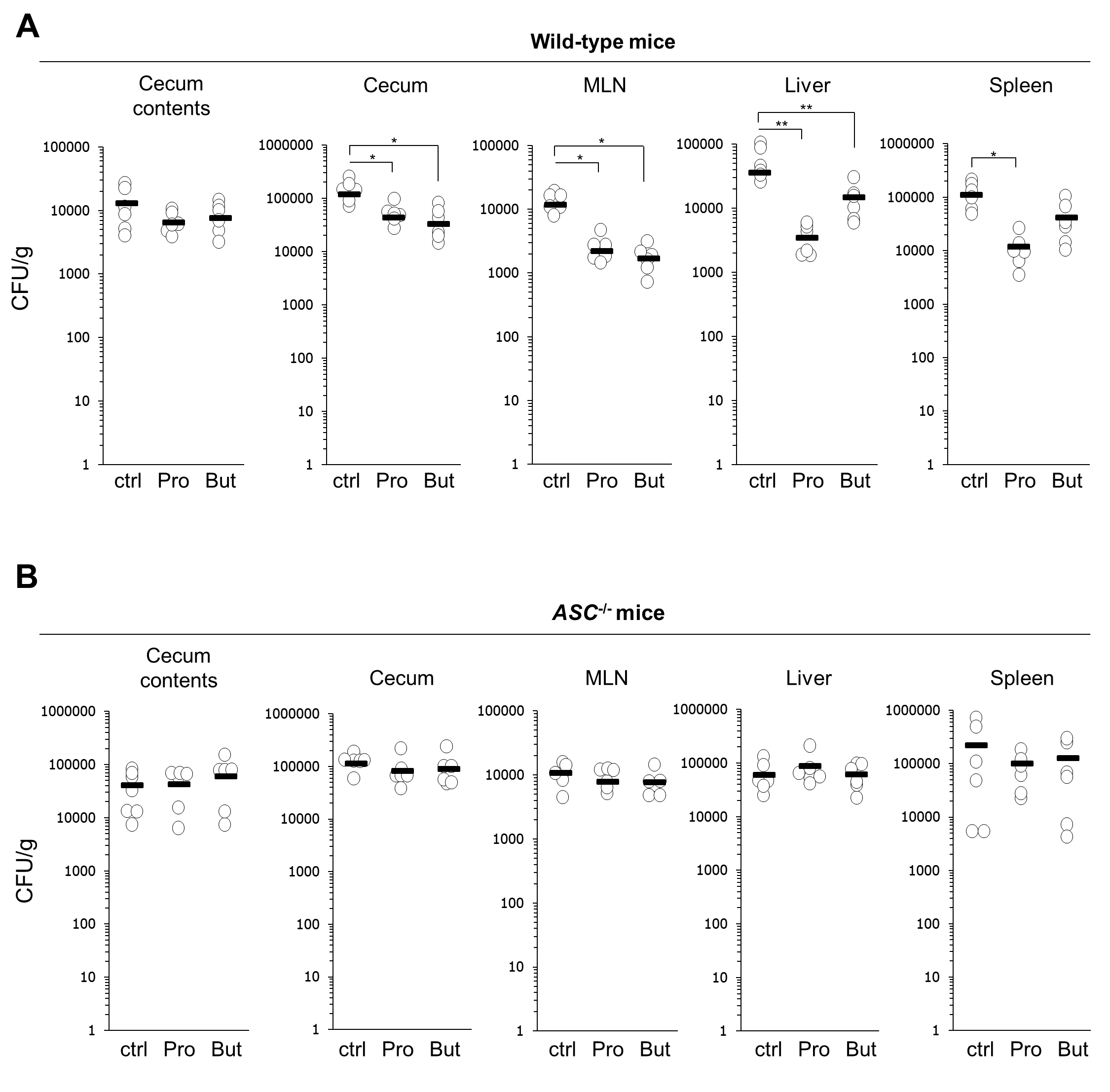

Supplement: S11 Fig — (A and B) Bacterial cells in the cecum contents, cecum tissues, MLN, liver, and spleen were counted 2 days after infection. Cecum contents and each tissue were homogenized in PBS, and then the homogenates were plated on the Salmonella Shigella selection agar (Difco SS agar) and colony-forming units were counted. Bars indicate the mean (n = 6 per group). One-way ANOVA analysis, *P < 0.05, **P < 0.01. Data are listed in S1 Data. ANOVA, analysis of variance; ASC, apoptosis-associated speck-like protein; MLN, mesenteric lymph node; S. Typhimurium, S. enterica serovar Typhimurium. (TIF) [file pbio.3000813.s011.tif]

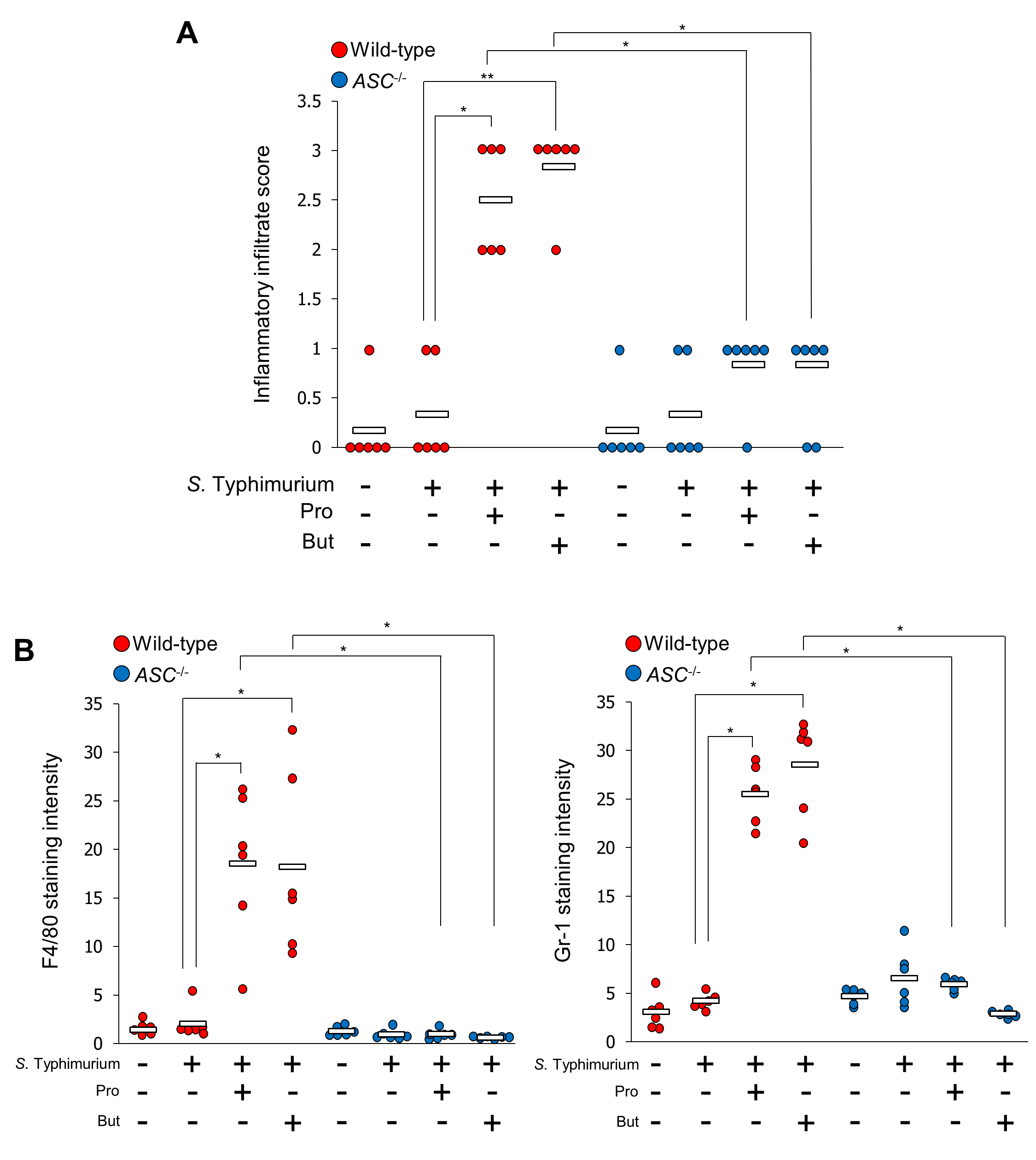

Supplement: S12 Fig — (A) The inflammatory infiltrate scores of HE-stained specimens were calculated. Bars indicate the mean (n = 6 per group). One-way ANOVA analysis, *P < 0.05, **P < 0.01. Data are listed in S1 Data. (B) The staining intensities for F4/80 and Gr-1 in the S. Typhimurium–infected cecum tissues of wild-type or ASC−/− mice were quantified using ImageJ analysis software. Bars indicate the mean (n = 6 per group). One-way ANOVA analysis, *P < 0.05. Data are listed in S1 Data. ANOVA, analysis of variance; ASC, apoptosis-associated speck-like protein; HE, hematoxylin–eosin; S. Typhimurium, S. enterica serovar Typhimurium. (TIF) [file pbio.3000813.s012.tif]

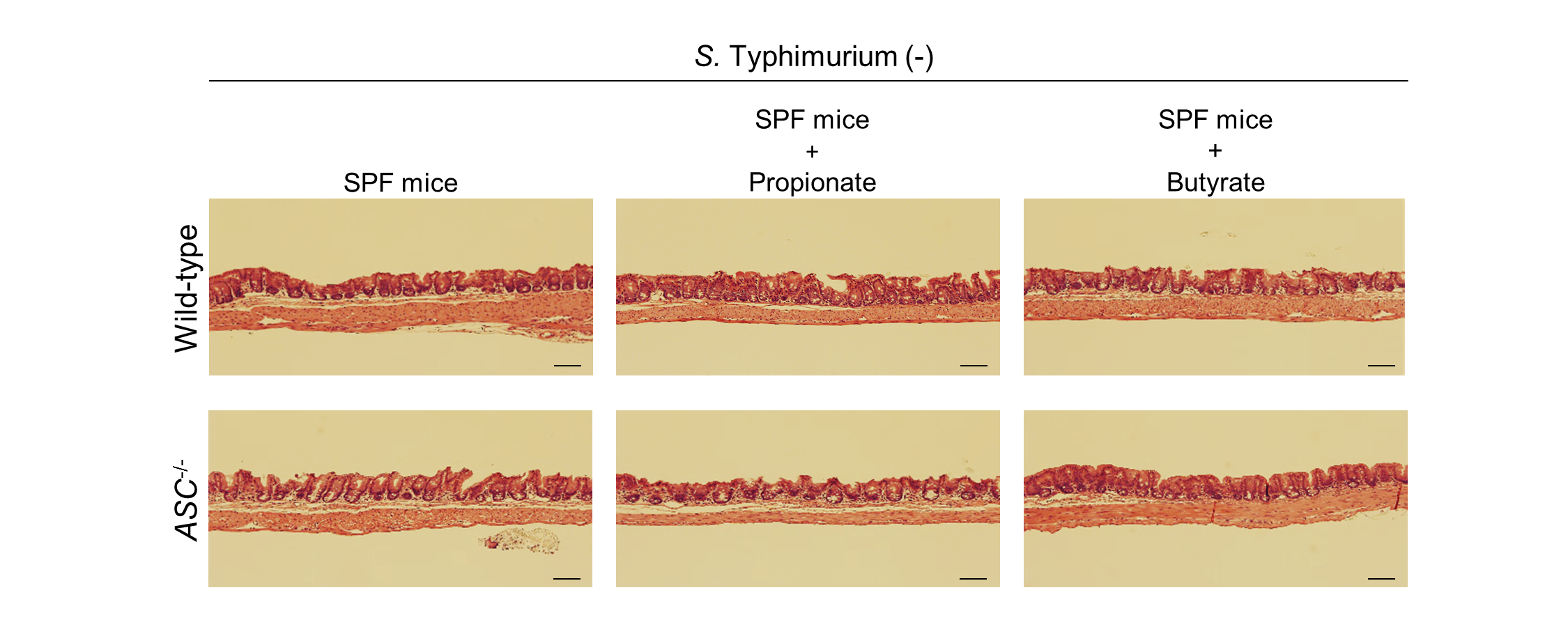

Supplement: S13 Fig — HE staining of cecum tissues from SCFAs-treated SPF wild-type or SPF ASC−/− mice without S. Typhimurium infection. Scale bars = 100 μm. ASC, apoptosis-associated speck-like protein; HE, hematoxylin–eosin; S. Typhimurium, S. enterica serovar Typhimurium; SCFA, short-chain fatty acid; SPF, specific pathogen-free. (TIF) [file pbio.3000813.s013.tif]

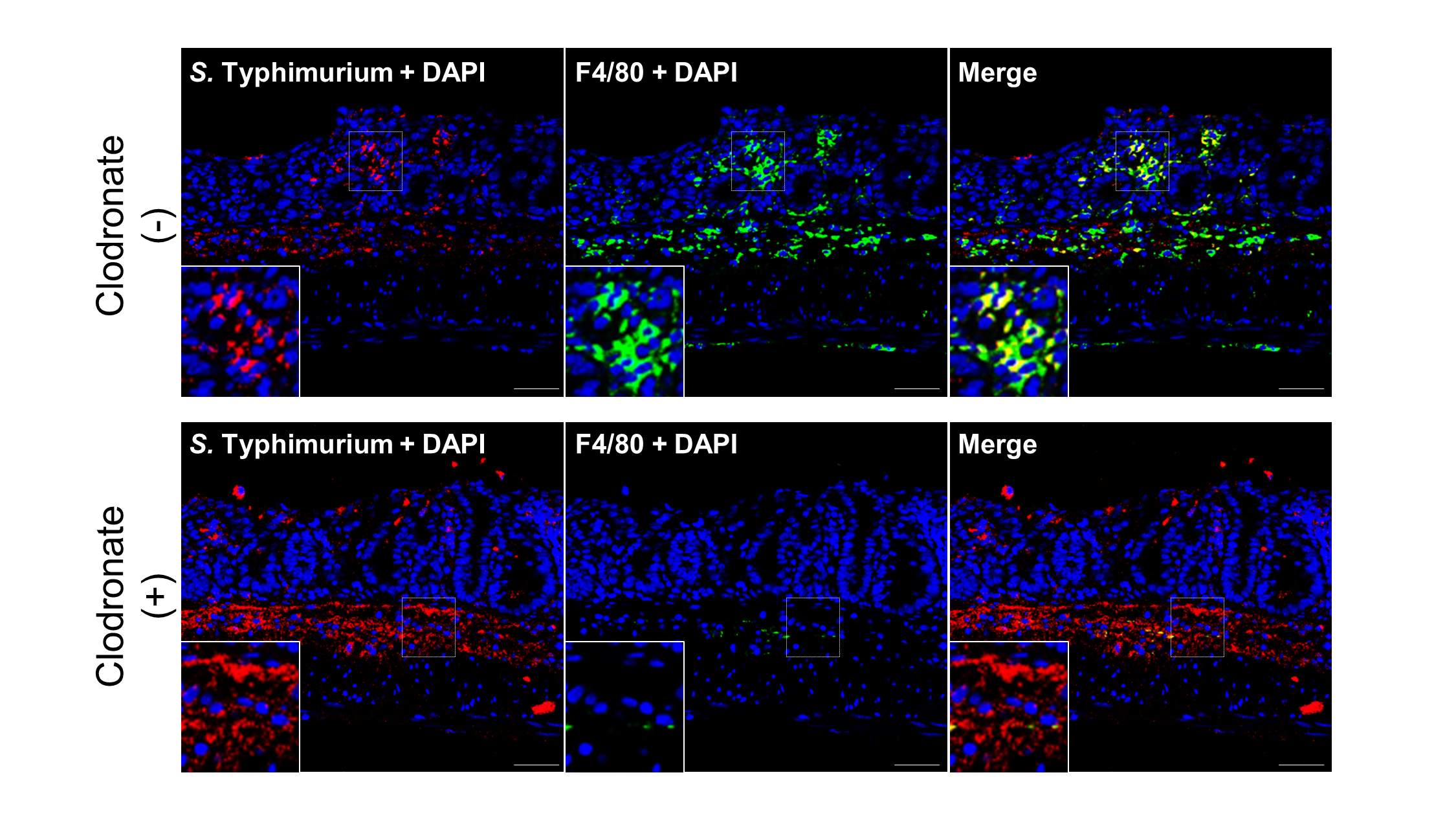

Supplement: S14 Fig — Immunostaining of cecum tissues using an anti-F4/80 antibody and an anti–S. Typhimurium LPS antibody. Scale bars = 50 μm. S. Typhimurium, S. enterica serovar Typhimurium. (TIF) [file pbio.3000813.s014.tif]

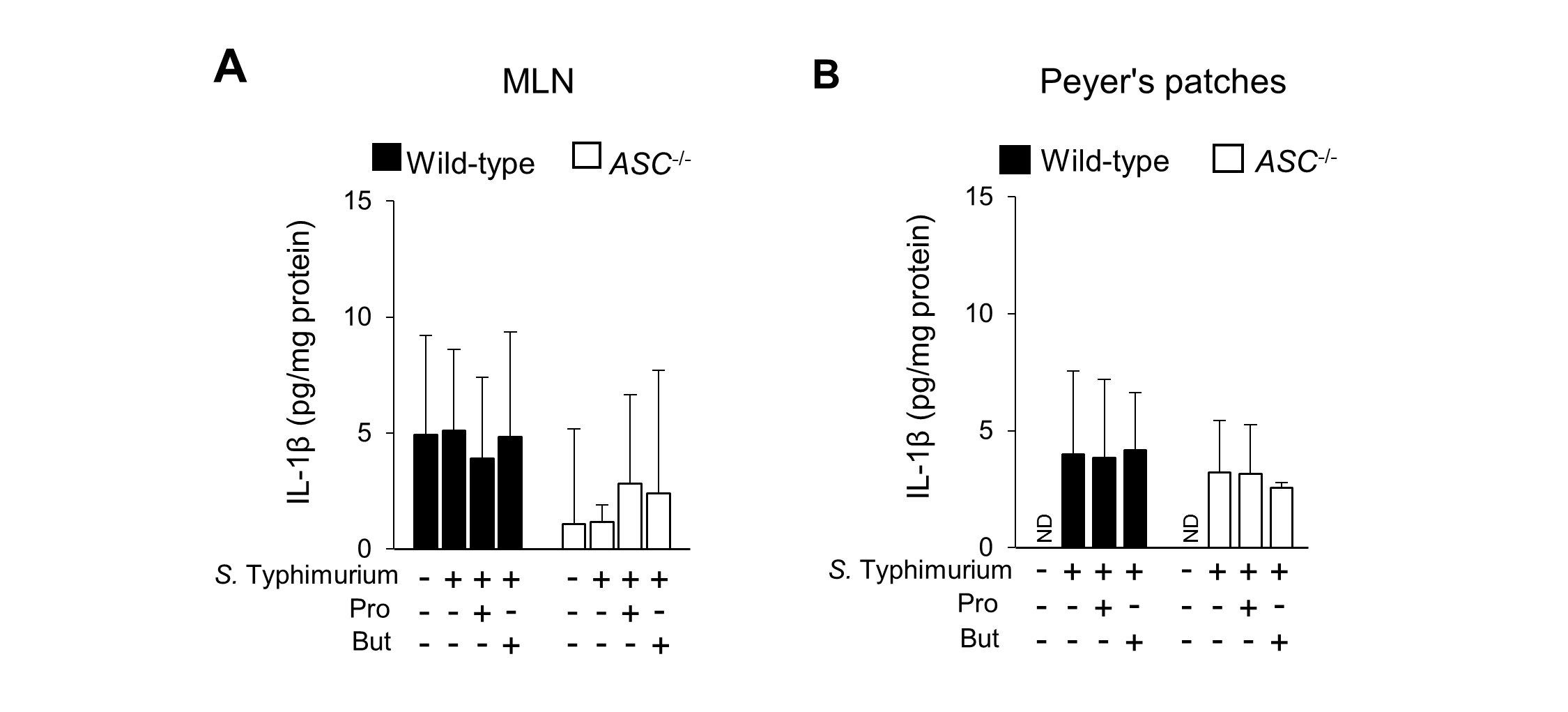

Supplement: S15 Fig — (A) IL-1β production in MLN as determined by ELISA (n = 4 per group). (B) IL-1β production in Peyer’s patches as determined by ELISA (n = 4 per group). Data are listed in S1 Data. ASC, apoptosis-associated speck-like protein; IL, interleukin; MLN, mesenteric lymph node; ND, not detected (below the detection limit). (TIF) [file pbio.3000813.s015.tif]
