# Supplementary material for: Short-chain fatty acids bind to apoptosis-associated speck-like protein to activate inflammasome complex to prevent Salmonella infection
Source: PLoS Biol. 2020 Sep 29;18(9):e3000813. doi: 10.1371/journal.pbio.3000813 (PMC7524008; doi:10.1371/journal.pbio.3000813)
Supplement: Attachment — Submitted filename: Point-by-Point response to reviews(PBIOLOGY-D-20-00270R1).docx [file pbio.3000813.s017.docx]

**Reviewer #1:** *Short chain fatty acids have been of continued interest due to their various effects on the microbiota and the host. The presented study by Tsugawa et al. sheds light on the mechanism of how SCFAs modulate the immune response and prolong survival after Salmonella infection. The authors determined that SCFAs bind to a lysine-rich domain of ASC, which facilitates binding to NLRPs. Macrophages exposed to SCFAs killed Salmonella more efficiently in vitro. In vivo, SCFA administration to antibiotics-treated mice increased their survival. No increased survival was seen for SCFA administration to ASC^−/−^ mice. Administration of soluble fiber increased survival of Salmonella-infected mice.*

*This manuscript uncovers an interesting mechanism that should be of interest to a broad audience, as SCFA-mediated enhancement of the immune response will likely occur also with other intestinal pathogens. The experiments were in general performed rigorously and include important controls. The authors used a variety of different methods, including imaging mass spectrometry, to support their hypothesis. However, for some experimental assays, additional information and clarifications need to be provided.*

*The manuscript has two main weaknesses: 1) co-housing instead of using littermates and the lack of microbiota analysis and 2) no experimentation to exclude that added SCFAs regulate virulence gene expression in Salmonella.*

*Co-housing apparently rendered the observed phenotype in Fig. 1 less pronounced. The microbiota analysis would, especially as fiber fermentation is subject of analysis, be a valuable addition to the paper. It could help dispel concerns that phenotypes are due to microbiota difference not resolved during co-housing. A number of studies have shown that SCFAs modulate Salmonella virulence gene expression. This was not addressed experimentally or discussed.*

**(Response)**

We appreciate these critical comments and insightful suggestions, which have helped us to considerably improve our paper. In the revised version of the manuscript, we have taken all of these suggestions on board. First, to overcome concerns that differences in phenotypes resulted from co-housing wild-type and *ASC*^−/−^ mice, we conducted an additional experiment to compare the gut microbiota of these mice. Second, to clarify the involvement of the expression of *Salmonella* pathogenicity island 1 (SPI-1), a virulence factor known to be regulated by SCFAs, in the enhanced inflammasome activation observed in response to SCFA treatment, we conducted additional experiments to evaluate the effect of SCFAs in mice infected with a mutant strain of *S*. Typhimurium lacking expression of SPI-1. We also discuss the effect of SPI-1 expression on the enhanced inflammasome activation by SCFAs in the revised manuscript. Detailed responses to specific comments from the reviewer are provided below.

***Comment 1.*** *Include analysis of the microbiota to show that WT and ASC^−/−^ mouse microbiota is highly similar before the start of the experiment and during recovery. If there are differences before, differences in the microbiota that recovers after the stop of antibiotic treatment could be partially responsible for the observed phenotype.*

**(Response)**

Thank you for this important suggestion. We conducted a 16S rRNA metagenome analysis of the gut microbiota to compare the gut microbiota between wild-type and *ASC*^−/−^ mice. There was no significant difference in the diversity of gut microbiota between wild-type and *ASC*^−/−^ mice before the administration of the antibiotics (S8 Fig). This suggests that the difference in susceptibility to *S.* Typhimurium between wild-type and *ASC*^−/−^ mice depends on ASC, not on a difference in the microflora recovery after antibiotic treatment. These data are shown as Supplementary Figure 8 of the revised manuscript, and the Results and Methods sections have been revised to include these data, as follows:

Line 273: To compare the microbiota in wild-type and *ASC*^−/−^ mice, the 16S rRNA metagenomes of gut microbiota from wild-type and *ASC*^−/−^ mice were analyzed. There was no significant difference in the diversity of gut microbiota between wild-type and *ASC*^−/−^ mice (S8 Fig). This result suggests that the increased susceptibility of SPF *ASC*^−/−^ mice to *S.* Typhimurium depends directly on ASC, not on a difference in the microflora.

Line 610: **16S rRNA sequencing of microbiota and profile analysis.** Fresh fecal samples were collected from five wild-type or *ASC*^−/−^ mice, and genomic DNA was extracted using the NucleoSpin Microbial DNA Kit (TaKaRa, cat. no. U0235) according to the manufacturer’s instructions. The extracted genomic DNA was sent to Macrogen Corp. (Tokyo, Japan) for metagenomics sequencing. The sequencing library was prepared by random fragmentation of the DNA, followed by 5' and 3' adapter ligation using Herculase II fusion DNA polymerase (Agilent, cat. no. 600675) and Nextera XT Index Kit V2 (Illumina, cat. no. FC-131-1001), according Illumina’s guidelines for 16S metagenomic sequencing library preparation (Illumina, Part #15044223 Rev. B). The amplicon of 16S rRNA was sequenced using paired-end sequencing on the Illumina MiSeq platform. The sequences were clustered *de novo* into Operational Taxonomic Units (OTUs) using a CD-HIT-EST-based OTU analysis program (CD-HIT-OTU) and a cut-off of >97% similarity. The Shannon diversity index and the Inverse Simpson index, which account for both richness and evenness, and where an increased value indicates greater diversity, were used to compare the microbial diversity of the OTUs in the libraries.

***Comment 2.*** *Related to point 1: The experimental setup in 4B is unclear. Please indicate with horizontal bars the periods when the antibiotic cocktail was administered and when SCFAs were administered. Was administration of the cocktail stopped before Salmonella infection or was a resistant Salmonella strain used?*

**(Response)**

Thank you for these comments. According to your suggestion, we added three horizontal bars to Fig. 4B indicating the period of antibiotic administration, the period of SCFA administration, and the period of *S*. Typhimurium infection. This revision should make it clear that the administration of the antibiotic cocktail was stopped before *S*. Typhimurium infection. In addition, the Materials and Methods and Figure Legends have been revised as follows:

Line 783: The administration of antibiotics was stopped before bacterial infection.

Line 965: (B) Treatment scheme followed to analyze the effect of SCFA administration on the susceptibility of antibiotic-treated mice to *S*. Typhimurium strain A. SPF mice were administrated antibiotics for 4 weeks. SCFAs were given 1 week before *S*. Typhimurium strain A infection. The administration of antibiotics was stopped before *S*. Typhimurium strain A infection.

***Comment 3.*** *The authors did not indicate for how long they co-housed the mice. Extensive co-housing is accepted by researchers as a way to normalize the microbiota of strains of different origins. However, the authors used male mice for their experiments, where co-housing would presumably result in severe aggression. Please indicate for how long mice were co-housed and how they were housed during experiments (single vs. group).*

**(Response)**

Thank you for this comment. In our *in vivo* experiments, both male and female C57BL/6J mice were used, and, to match the gender, C57BL/6J male mice were purchased from SLC Japan. In co-housing experiments, SPF mice were co-housed at a 1:1 gender ratio for 7 days. The period of co-housing followed a previous study (***Nature***, 501; 112–115, 2013). This information has been added to the Materials and Methods and Figure Legends, as follows:

Line 592: Male and female wild-type, *ASC*^−/−^, and *GPRR43*^−/−^ mice were used for experiments. To match the gender in the experiment, six-week-old C57BL/6J male mice were purchased from SLC Japan (Shizuoka, Japan).

Line 770: SPF wild-type mice were co-housed with SPF *ASC*^−/−^ mice at a 1:1 gender ratio for 7 days prior to *S*. Typhimurium infection (***Nature***, 501; 112-115, 2013).

Line 960: (A) SPF wild-type mice were co-housed with SPF *ASC*^−/−^ mice at a 1:1 gender ratio for 7 days, and then were infected with *S*. Typhimurium strain A.

***Comment 4.*** *SCFAs like butyrate and propionate are known to down-regulate Salmonella SPI-1 expression at higher concentrations. Experiments with ASC^−/−^ mice and BMDM seem to indicate that this is not a relevant mechanism in this model. No actual numbers, just percentages are given, so it is difficult to interpret. A probably relevant control would be an experiment with a SPI-1 deficient Salmonella strain and WT and ASC^−/−^ BDMDs.*

**(Response)**

Thank you for this important comment. To examine the involvement of SPI-1 in the enhanced inflammasome activation by SCFAs, an *S*. Typhimurium SPI-1 knock-down mutant strain was used. These data are shown in Supplementary Figure 6 of the revised manuscript.

Lawhon *et al*. showed that SCFAs at a concentration of 30 mM decreases the expression of *Salmonella* SPI-1 genes *in vitro* (***Mol. Microbiol.***, 46; 1451-64, 2002). We showed that SCFAs at a concentration of 10 mM enhanced the elimination of *S*. Typhimurium in BMDMs by promoting inflammasome activation through ASC-dependent mechanisms. Therefore, we examined the effect of SCFAs at a concentration of 10 mM on the SPI-1 gene expressions in *S*. Typhimurium strain A. The expression of SPI-1 genes (*sipD* and *prgH*) did not change in response to treatment with 10 mM propionate, 10 mM butyrate, or 10 mM lactate (S6A Fig), indicating that the enhanced elimination of *S*. Typhimurium in BMDMs by 10 mM SCFAs is independent of the down-regulation of SPI-1 genes *in vitro*.

We also evaluated the effect of SCFAs on inflammasome activation in BMDMs using an *S*. Typhimurium SPI-1 knock-down strain. The SPI-1 knock-down strain of *S*. Typhimurium was constructed by introducing an in-frame deletion of *sipB*, a transcriptional activator of SPI-1, and we confirmed that the expression of SPI-1 genes (*sipD* and *prgH*) was significantly repressed in the *S*. Typhimurium SPI-1 knock-down strain (S6A Fig).

When using the SPI-1 knock-down strain of *S*. Typhimurium, the infection number in BMDMs and the induced LDH release were similar in wild-type and ASC knockout, and only a weak effect of SCFAs was observed in wild-type BMDMs (S6B and S6C Fig). On the other hand, the IL-1β production from wild-type BMDMs infected with SPI-1 knock-down *S*. Typhimurium was significantly increased by treatment with 10 mM propionate or 10 mM butyrate, but this was not the case in *ASC*^−/−^ BMDMs (Fig. S6D). These results suggest that SCFAs can enhance the production of IL-1β through an ASC-dependent mechanism in macrophages infected with SPI-1 knock-down *S*. Typhimurium, but the pyroptosis associated with the elimination of the *S*. Typhimurium SPI-1 knock-down strain is independent of ASC. These data have been added to the revised manuscript as Supplementary Figure 6, and included in the revised Results sections and Materials and Methods sections, as follows:

Line 571: **Construction of the *S*. Typhimurium SPI-1 knock-down mutant strain.** SPI-1 knock-down bacteria were constructed by introducing an in-frame deletion of *sipB*, a transcriptional activator of SPI-1. A FRT (Flp recombination target)-flanked chloramphenicol-resistant marker cassette (KD3) fragment was amplified using the primers 5'-GTGTAGGCTGGAGCTGCTTC-3' (forward) and 5'-ATGGGAATTAGCCATGGTCC-3' (reverse) (***J Med Microbiol.***, 64; 295-302, 2015), and then the PCR products were inserted into pGFPmut 3.1 (Clonetech, cat. no. 632370) downstream of *gfp*. The *sipB*::*gfp*-KD3 fragment was amplified using the following primers:

5'-CGGAGACAGAGCAGCACAGTGAACAAGAAAAGGAATAATTATGCGTAAA

GGAGAAGAACT-3' (forward) and 5'-TACTAATTAACATATTTTTCTCCCTTTATT

TTGGCAGTTTGTGTAGGCTGGAGCTGCTTC (reverse).

The PCR product of the *sipB*::*gfp*-KD3 fragment was introduced into *S*. Typhimurium SR-11 χ3306/pKD46 by electroporation to construct the *sipB* in-frame replacement strain of *S*. Typhimurium SR-11 χ3306. The in-frame *sipB* deletion on the chromosome of *sipB*::*gfp*-KD3 fragment-electroporated *S*. Typhimurium SR-11 χ3306 was accomplished by λ Red-mediated recombination (***PNAS***, 97; 6640-6645, 2000).

***Comment 5.***　*Are the pictures in Fig. 5B truly representative? The immune cell recruitment with SCFAs is massive. Fig. 5C shows more moderate recruitment. Please provide pathology scores to assess all and not just representative mice.*

**(Response)**

Thank you for this important suggestion. We re-evaluated these data by calculating the inflammatory infiltrate score and the staining intensities of F4/80 and Gr-1 (n = 6 mice per group), and these data were added to the revised manuscript as Supplementary Figure 12. The inflammatory infiltrate score of H&E-stained tissues was calculated according to the method published by Koelink *et al*. (***J. Crohns Colitis***, 12; 794-803, 2018). The inflammatory infiltrate scores of *S*. Typhimurium-infected wild-type mice administered SCFAs were significantly higher than those of mice that did not receive SCFAs or of *S*. Typhimurium-infected *ASC*^−/−^ mice (S12A Fig). In addition, the staining intensities of F4/80 and Gr-1 in the *S*. Typhimurium-infected cecum tissues of wild-type or *ASC*^−/−^ mice were quantified using ImageJ software (n = 6 mice per group). The staining intensities of F4/80 and Gr-1 in the cecum tissues of *S*. Typhimurium-infected wild-type mice administered SCFAs were significantly higher than in *S*. Typhimurium-infected wild-type mice without SCFAs or in *S*. Typhimurium-infected *ASC*^−/−^ mice (S12B Fig). These data are included in the revised manuscript as Supplementary Figure 12, and the Results section has been revised as follows:

Line 322: The inflammatory infiltrate score of the H&E-stained tissues was calculated according to a previously published method (***J. Crohns Colitis***, 12; 794-803, 2018). The inflammatory infiltrate scores in *S*. Typhimurium-infected wild-type mice administrated with propionate or butyrate were significantly higher than in mice that had not been treated with SCFAs or in *S*. Typhimurium-infected *ASC*^−/−^ mice (S12A Fig).

Line 335: The staining intensities of F4/80 in the cecal mucosa and submucosa of *S*. Typhimurium-infected wild-type mice administrated with propionate or butyrate were significantly higher than in *S*. Typhimurium-infected wild-type mice without SCFA treatment or in *S*. Typhimurium-infected *ASC*^−/−^ mice (S12B left panel Fig).

Line 344: The staining intensities of Gr-1 in the cecal mucosa and submucosa of *S*. Typhimurium-infected wild-type mice which were orally given propionate or butyrate were significantly higher than in *S*. Typhimurium-infected wild-type mice without SCFA treatment or in *S*. Typhimurium-infected *ASC*^−/−^ mice (S12B right panel Fig).

***Comment 6.*** *Were all experiments done exclusively with male mice? The purchased C57BL/6 mice were male, but no indication for other strains is given. Ideally (and most often now requested) experiments should be performed with male and female mice to exclude gender bias.*

**(Response)**

Thank you for the comment. Both male and female wild-type, *ASC*^−/−^, and *GPRR43*^−/−^ mice were used. To match the genders, male C57BL/6J mice were purchased from SLC Japan. The Materials and Methods section has been revised to include this information, as follows:

Line 592: Male and female wild-type, *ASC*^−/−^, and *GPRR43*^−/−^ mice were used for experiments. To match the gender in the experiment, six-week-old C57BL/6J male mice were purchased from SLC Japan (Shizuoka, Japan).

**Minor comments:**

***Comment 1.*** *Manuscript is well written, but it needs to be thoroughly checked (e.g. "% of survive" or "infection to wild-type mice" and "scarify" in Fig. 4).*

**(Response)**

Thank you very much for comment. We have corrected some words and phrase according to your comment as follow:

The phrase "% of survive" has been corrected to "Survival (%)". The phrase "infection to" has been deleted and revised in Fig. 4, 7, and S7. The word "scarify" has been corrected to "sacrifice".

***Comment 2.*** *Multiple mouse models exist for Salmonella Typhimurium. Please provide the rationale for the choice of the mouse model you use (e.g. lower colonization and cecum inflammation than streptomycin pre-treatment mouse model).*

**(Response)**

Thank you for the comment. In the present study, to clarify the role of SCFAs in protection against *S*. Typhimurium infection, we needed to exclude the effects of endogenous SCFAs derived from gut commensal bacteria. In Rakoff-Nahoum *et al*.'s report, the combination therapy of four antibiotics (ampicillin, metronidazole, neomycin, and vancomycin) was used for mice for complete depletion of gut commensal bacteria, and the effect of this combination therapy was verified by bacteriologic analysis using colonic feces (***Cell***, 118; 229–241, 2004). Therefore, to completely eradicate gut commensal bacteria, mice were administered this combination of four antibiotics for 4 weeks prior to *S*. Typhimurium infection. Propionate and butyrate in the cecal lumen were completely deceased by this combination treatment regimen (S9 Fig). Our rationale for choosing this mouse model has been added to the Materials and Methods section, as follows:

Line 771: To clarify the role of SCFAs in the protection against *S*. Typhimurium infection, it was important to exclude the effects of endogenous SCFAs by completely eradicating gut commensal bacteria. Rakoff-Nahoum *et al*. reported that the combination therapy of four antibiotics (ampicillin, metronidazole, neomycin, and vancomycin) for mice was used for complete depletion of gut commensal bacteria (***Cell***, 118; 229–241, 2004). Therefore, to exclude the effects of endogenous SCFAs derived from gut microbiota, 8-to-10-week-old mice were given drinking water containing ampicillin (1 g/L; Sigma-Aldrich, cat. no. A0166), metronidazole (1 g/L; Sigma-Aldrich, cat. no. M1547), neomycin (1 g/L; Sigma-Aldrich, cat. no. N1876), and vancomycin (0.5 g/L; Wako, cat. no. 222-01303) for 4 weeks prior to *S*. Typhimurium infection, as previously described (***Cell***, 118; 229-241, 2004, ***Immunity***, 29; 637-649, 2008).

**Comment 3.**　M&M clarifications needed:

*1, Fiber and composition of the diet is important for the observed phenotype. Please indicate which diet the mice were fed.*

**(Response)**

Thank you for the comment. We used a standard rodent diet, the CE-2 diet, which consists mainly of vegetable protein (soybean meal) and animal protein, and contains a fiber source (wheat bran, defatted rice bran, and alfalfa meal). This information has been added to the Materials and Methods sections, as follows:

Line 589: All mice were fed the CE-2 diet (Japan Clea, Tokyo, Japan), which consists mainly of vegetable protein (soybean meal) and animal protein, and contains a fiber source (wheat bran, defatted rice bran, and alfalfa meal).

*2,* *Mice are known to avoid drinking the antibiotic cocktail. How much weight did they lose before the beginning of the experiment? This should be noted to orient the reader to the fact that there are more differences between SPF and antibiotics-treated mice than the presence or absence of bacteria.*

**(Response)**

Thank you for comment. In our experiments, most mice did not lose weight, but about 4.2% of mice (1 in 24) lost more than 20% of their weight during the antibiotic administration period. Following the guidelines for animal care and use of the Keio University Animal Research Committee, these mice were not used in our experiments and were humanely euthanized. This information has been added to the Materials and Methods section of the manuscript, as follows:

Line 781: Mice that lost more than 20% of their body weight by refusing to drink the antibiotic cocktail were excluded from experimental use, following the guidelines of the Keio University Animal Research Committee.

*3, The Salmonella strain used is likely ATCC 14028S and not ATCC 14208S?*

**(Response)**

Thank you for pointing out the details. We apologize for the error. We have corrected "ATCC 14208S" to "ATCC 14028S" in lines 266, 268, 565, and 568, and in Supplementary Figure 7.

*4, Rationale for using U937 cells, mention also in results.*

**(Response)**

Thank you for this comment. U937 cells have been used to study the activity and differentiation of monocytes by many researchers. In addition, U937 cells are known to exhibit the morphology and characteristics of mature macrophages, and are able to respond to many exogenous stimuli. Our purpose in this study was to identify authentic SCFA receptors in immune cells, in order to evaluate their protective mechanisms against pathogenic infection. We therefore used U937 cells to identify novel SCFA-binding proteins using SCFA-conjugated beads. We have added this rationale for using U937 cells to the Results section, as follows:

Line 131: Human U937 lymphoma cells are known to exhibit the morphology and characteristics of mature macrophages and are able to respond to many exogenous stimuli. To identify authentic SCFA receptors in immune cells in order to evaluate their role in protection against pathogenic infection, we conducted a binding assay using SCFA-conjugated beads and the soluble (cytosolic) fraction of U937 cells.

*5, Some key information, e.g. how long BMDMs were incubated with Salmonella, is only available in the figure legend and not in the M&M. Please add where appropriate.*

**(Response)**

Thank you for your comment. The Materials and Methods section has been revised to include these details, as follows:

Line 729: BMDMs were incubated with *S*. Typhimurium strain A at a multiplicity of infection of 5 for 10 min, and then washed three times with PBS and incubated in DMEM containing 100 μg/mL gentamycin for 15 hours with or without SCFAs at a concentration of 1–10 mM.

***Comment 4.*** *Fig. 3: Percent of LDH release is compared to which positive control?*

**(Response)**

Thank you for comment. The cells were treated with 0.8% TritonX-100 as a positive control. The percent LDH release was calculated as the ratio of the amount of LDH released in each experimental condition to the amount of LDH released by cells treated with 0.8% TritonX-100, as follows: % LDH release = [experimental LDH release (OD_490_) − average LDH release in the presence of culture medium (OD_490_)]/[LDH release in cells treated with 0.8% TritonX‐100 (OD_490_) – average LDH release in the presence of culture medium (OD_490_)] × 100. This calculation has been added to the Materials and Methods section of the revised manuscript:

Line 738: The percent LDH release was calculated as the ratio of the amount of LDH released under each experimental condition to the amount of LDH released by cells treated with 0.8% TritonX, as follows: % LDH release = [experimental LDH release (OD_490_) − average LDH release in cells treated with culture medium (OD_490_)]/[LDH release in cells treated with 0.8% TritonX-100 (OD_490_) − average LDH release in cells treated with culture medium (OD_490_)] × 100.

***Comment 5.*** *Fig. 7: Imaging mass spectrometry is a very useful tool to illustrate the penetration of SCFAs into the tissue. In uninfected mice, the cecal tissue is easily identified, and also in sections of the infected mice. However, there seems to be absolutely no structure in the remaining parts, rather only huge rips. Are these artefacts from processing? Please explain this a bit more.*

**(Response)**

Thank you for the comment. The rips in *S*. Typhimurium-infected cecal tissues were caused by experimental processing. Sections of frozen and embedded caecum tissues are prepared with a cryomicrotome, and then the sections are attached to indium tin oxide-coated glass slides. *S*. Typhimurium-infected cecal mucosa and submucosa loses some of its structural stability due to the inflammation. These rips occur more easily in the processing of frozen sections compared with paraffin-embedded sections. However, the boundary between the cecal lumen and the mucosa could be identified despite the tears. A dotted line has been added to the images to mark this boundary. This explanation has been added to the Results section, as follows:

Line 414: *S*. Typhimurium-infected cecal tissues lose structural stability due to the inflammation, resulting in some tearing of the cecal mucosa and submucosa during the preparation of frozen sections. Despite this, the boundary between the cecal lumen and the mucosa could be identified in these sections, and is indicated by a dotted line.

***Comment 6.***　*Fig. 3E: Why are here CFU/ml and not CFU as percent of control? How did this assay differ from the other similar assays in Fig. 3?*

**(Response)**

Thank you for these comments. We apologize for the confusion. Fig. 3A and Fig. 3E are the same assay. We have revised Fig. 3E to show the data in the same format as in Fig 3A, where the CFU is expressed as a percent of the control.

***Comment 7.***　*Fig. 3D: Please indicated that levels were below limit of detection and are not missing data.*

**(Response)**

Thank you for the comment. It is correct that in Fig. 3D, the values represented by “ND” were below the limit of detection. This information has been added to the legend for Fig. 3D, as follows:

Line 951: ND, not detected (below the detection limit).

***Comment 8.***　 *Fig. S4E: I assume you have done multiple tests to confirm the deletion, but this WB does not look convincing.*

**(Response)**

Thank you for the comment. To confirm the deletion of *ASC* or *GPR43*, we conducted an additional PCR analysis of genomic DNA isolated from each mouse strain. These data confirm the deletion of *ASC* or *GPR43* in genomic DNA isolated from *ASC*^−/−^ or *GPR43*^−/−^ mice, respectively. These data are shown in Supplementary Figure 4 of the revised manuscript, and the Materials and Methods and Figure Legends have been revised, as follows:

Line 596: To confirm the deletion of *ASC*, a 350 bp fragment of the *ASC* gene was amplified from genomic DNA using the primers 5'-CCAGGTAATGGTTAATCCCAGCAACC-3' (forward) and 5'-TTGGCATTGCATGACATGGTGCACAC-3' (reverse).

Line 605: To confirm the deletion of *GPR43*, a 390 bp fragment of the *GPR43* gene was amplified from genomic DNA using the primers 5'-CAGACTGGCACAGTTCCTTGATCC-3' (forward) and 5'-GCGATCACTCCATACAGTGGCC-3' (reverse).

***Comment 9.*** *Fig. S7: Why was the extra SCFA boost necessary? Lines 252-254 seems to indicate that SCFA administration in the drinking water increases SCFA levels to similar levels as in SPF mice. However, the authors seem to measure an undefined boost and not what is available during Salmonella infection.*

**(Response)**

Thank you for this comment. In our *in vivo* experiments, exogenous SCFAs were administered to antibiotic-treated mice in the drinking water. An experimental limitation of this method is that it is difficult to determine the exact concentration of the SCFAs in the cecal lumen of the mice because the timing and amount of water intake are different in each mouse, resulting in inconsistent data. To measure the concentration of SCFAs in the cecal lumen immediately after exogenous SCFA administration, cecum samples were collected following an oral boost with 100 μL of 300 mM propionate, 300 mM butyrate, and 300 mM lactate 2 h prior to sacrifice. The Results and Materials and Methods sections of the manuscript have been revised to include this explanation, as follows:

Line 285: Treatment with antibiotics significantly reduced the concentrations of propionate and butyrate in the cecal lumen (S9 Fig). To measure the concentration of SCFAs in the cecal lumen of the antibiotic-treated mice immediately after exogenous administration of SCFAs, the mice were boosted orally with 100 μL of 300 mM propionate or butyrate 2 h prior to sacrifice, and the SCFA concentrations in the cecal lumen were measured. The exogenous administration of SCFAs restored their concentrations to the levels observed in SPF mice not treated with antibiotics (S9 Fig).

Line 786: Since exogenous SCFAs were administrated to antibiotic-treated mice in the drinking water, the concentrations of SCFAs in the cecal lumen were variable at the time of sacrifice depending on the length of time since water intake. Therefore, the concentrations of SCFAs in the cecal lumen of mice were measured after an oral boost with 100 μL of 300 mM propionate or butyrate 2 h prior to sacrifice.

***Comment 10.*** *Fig. S9: Bacterial numbers recovered from mice usually have log-normal distribution and are best displayed on a log scale.*

**(Response)**

Thank you for this comment. Fig. 6 and S11 have been corrected to show the data on a log scale.

***Reviewer #2:*** *Tsugawa et al provide evidence for an interesting model in which short chain fatty acids (SCFAs), which are produced through fermentation of dietary fiber by members of the gut microbiota, facilitate the interaction between the inflammasome pattern recognition receptor Nlrp3 and its adaptor Asc, leading to enhanced inflammasome activation. The authors pinpoint a lysine rich region of the pyrin domain as important for SCFA binding. Furthermore, SCFA treatment of macrophages leads to pyroptosis and restricts Salmonella intracellular survival. In addition, SCFA administration to mice induces inflammation and enhances resistance against Salmonella infection. These results suggest the intriguing possibility that eating a diet rich in fiber may protect against Salmonella infection. However, the manuscript is lacking in sufficient context with respect to previous studies involving Salmonella and SFCAs. A number of references important to this work are omitted and not discussed.*

**(Response)**

We appreciate the reviewer’s critical comments and insightful suggestions, which helped us to considerably improve our paper. We have taken all these comments and suggestions into account in the revised manuscript.

First, according to the reviewer’s suggestion, we cited several important references in the Discussion section:

“While *S*. Typhimurium can metabolize butyrate by anaerobic β-oxidation due to its expression of the *ydiQRSTD* operon, which encodes metabolic enzymes related to β-oxidation of fatty acids (***Mol. Microbiol.***, 47; 793-805, 2003), exposure to a high concentration of butyrate directly represses the expression of *S*. Typhimurium invasion genes (*invA* and *hilD*) (***Appl. Environ. Microbiol.***, 72; 946-949, 2006, ***Mol. Microbiol.***, 46; 1451-1464, 2002, ***Cell Host Microbe***, 23; 266-273, 2018). Furthermore, Bronner *et al.* also showed that butyrate reduced *S.* Typhimurium colonization of Peyer’s patches by inducing down-regulation of these invasion genes (***Cell Host Microbe***, 23; 266-273, 2018). In our analyses, the IL-1β production in Peyer’s patches was not enhanced by butyrate, suggesting that butyrate does not enhance inflammasome activation in Peyer’s patches via an ASC-dependent mechanism (S15 Fig). These results support the idea that reduced *S.* Typhimurium colonization of Peyer’s patches depends on the down-regulation of invasion gene expression by butyrate. On the other hand, in the cecum, our results show that butyrate enhanced IL-1β production via an ASC-dependent mechanism (Fig. 5). While further study is needed to elucidate the precise role of invasion genes in bacterial colonization, our findings indicate that butyrate enhances inflammasome activation to eliminate the bacteria in the cecum.”

Second, to clarify the involvement of the *S*. Typhimurium virulence factor SPI-1 in the enhanced inflammasome activation by SCFAs, we conducted an additional experiment using an SPI-1 knock-down strain. Third, to examine whether administration of SCFAs to SPF mice without *S*. Typhimurium infection induces altered tissue architecture, we evaluated H&E-stained cecum tissues from SCFA-treated SPF wild-type or *ASC*^−/−^ mice. Finally, to assess the localization of *S*. Typhimurium in the cecal mucosa of mice administered clodronate-encapsulated liposomes, the *S*. Typhimurium-infected cecal mucosa was immunostained. Detailed responses to the reviewer’s comments are below.

***Comment 1.*** *There is no reference to PMID: 29447698 entitled "Genetic ablation of butyrate utilization attenuates gastrointestinal Salmonella disease". Among other things relevant to this manuscript, this paper showed that butyrate decreased Salmonella colonization of Peyer's patches in gnotobiotic mice.*

**(Response)**

Thank you very much for this important comment. We have added a discussion of these findings to the revised manuscript (see section quoted above).

***Comment 2.***　*If SCFAs induce ROS by neutrophils (pg 13 line 100), why would Salmonella colonization of the cecum decrease (Fig S9), as ROS induction by Salmonella itself leads to generation of alternative TEAs that allows Salmonella to outcompete microbiota (PMID: 27078066)?*

**(Response)**

Thank you very much for pointing out this important question. If SCFAs strongly induce excessive ROS production from neutrophils, gut commensal bacteria contributing to the control of colonization and/or expansion of *S*. Typhimurium might be eliminated (***Cell Host Microbe***, 19; 443-454, 2016; PMID: 27078066). Our results showed that enhanced IL-1β production by SCFAs promotes neutrophil infiltration of *S*. Typhimurium-infected cecal mucosa. Rapid neutrophil infiltration has been shown to efficiently eliminate and prevent the spread of *S*. Typhimurium (***Infect. Immun.***, 72; 468-477, 2004). Therefore, our results suggest that SCFAs promote the elimination of *S*. Typhimurium by enhancing neutrophil infiltration by promoting inflammasome activation in macrophages, in addition to inducing ROS production by neutrophils. We have revised the Introduction and Results sections to reflect this point, as follows:

Line 100: SCFAs reportedly not only promote the production of inflammatory mediators by macrophages, but also induce ROS production and phagocytosis by neutrophils (***Cell Biol. Int.***, 22; 331-337, 1998, ***Nutrients***, 3; 858-876, 2011). If SCFAs strongly induce excessive ROS production by neutrophils, gut commensal bacteria controlling the colonization and/or expansion of pathogens might be eliminated (***Cell Host Microbe***, 19; 443-454, 2016). The detailed effects of SCFAs on the function of immune cells with respect to protection against pathogens remain unknown. In particular, which molecules in immune cells react with SCFAs to regulate innate immunity remains incompletely understood, and authentic receptors for SCFAs must be identified to reveal their protective mechanisms against pathogen infection.

Line 352: While excessive ROS production by neutrophils might eliminate gut commensal bacteria that prevent the colonization and/or expansion of *S*. Typhimurium (***Cell Host Microbe***, 19; 443-454, 2016), rapid neutrophil infiltration efficiently prevents the spread of the pathogen (***Infect. Immun.***, 72; 468-477, 2004). Our results suggest that SCFAs promote the elimination of *S.* Typhimurium by efficiently promoting neutrophil infiltration of the cecal mucosa by enhancing inflammasome activation in macrophages.

***Comment 3.*** *What is the rationale behind using STm strain A and needing to compare it to ATCC 14028S?*

**(Response)**

Thank you for your comment. *S*. Typhimurium strain A is a clinical isolate that was obtained from the bacterial culture collection of the Research Institute for Microbial Diseases of Osaka University, Japan, and therefore we wanted to exclude the inherent pathogenicity of the *S*. Typhimurium strain A in mice. Accordingly, we compared the pathogenicity of *S*. Typhimurium strain A with that of the *S*. Typhimurium ATCC14028S strain before conducting *in vivo* infection experiments in mice. We have revised the Results sections to better clarify this point, as follows:

　　Line 264: To confirm that *S*. Typhimurium strain A is not inherent pathogenicity, we first compared the pathogenicity of *S*. Typhimurium strain A with that of the *S*. Typhimurium ATCC14028S strain in mice.

***Comment 4.*** *An explanation of how Nlrp3 normally gets activated and interacts with Asc is lacking. What's known and how could SCFAs fit in? Would SCFAs affect nucleation of Asc filaments by Nlpr3, as in Lu et al 2014 Cell (PMID: 24630722).*

**(Response)**

　　　Thank you for this comment. We have cited this report in the revised Discussion section, as follows:

Line 449: We showed that SCFAs interact with the Lys-rich region of the ASC PYRIN domain, which includes the Lys21 residue, suggesting that SCFAs bind to the basic region of the PYRIN domain and alter electrostatic interactions, allowing it to more easily interact with other PYRIN domains. Lu *et al*. reported that the PYRIN and CARD domains of ASC are both filaments, and that the PYRIN domain of ASC is nucleated by NLRP3, which senses PAMPs or DAMPs (***Cell***, 156; 1193-1206, 2014; PMID: 24630722). While further investigation is needed to elucidate whether SCFAs affect the nucleation of the PYRIN domain of ASC filaments, our findings suggest that SCFAs promote the interaction of NLRPs with ASC by binding to the Lys-rich region of the ASC PYRIN domain, leading to enhanced inflammasome activation to protect against *S*. Typhimurium infection.

***Comment 5.*** *Is it possible that the top band in Fig 1B found in the proprionate and butyrate samples but not the acrylate sample is polymerized Asc?*
**(Response)**

Thank you for this comment. These bands could be polymerized ASC, but this is not certain. In this study, we analyzed specific protein bands of around 25 kDa that bound to propionate and butyrate by ESI-MS. The Results section has been revised to clarify this, as follows:

Line 136: Peptide sequencing by electrospray ionization (ESI)-mass spectroscopy (MS) identified a protein with an apparent molecular weight of 25 kDa as the inflammasome adaptor protein ASC, after analyzing the protein bands that specifically bound to propionate and butyrate.

***Comment 6.***　*Fig 3A: What is shown in the images? If they are wells with infected monolayers, why are the cells growing in clumps? A scale bar would be useful.*

**(Response)**

Thank you for pointing out these items. We apologize for the confusion. Fig. 3A shows an LB agar plate cultured with *S*. Typhimurium isolated from macrophages for measurement of the CFU. This is a representative photographic result of an LB agar plate. In the revised version, these images have been removed from Fig. 3A to avoid confusion.

***Comment 7.***　*Why was procasp-1 and casp-1 precipitated out of the infected monolayer supernatant using acid rather than probed from cell lysate (Fig 3B)?*

**(Response)**

Thank you for the comment. The detection of the mature form of caspase-1 in the cytosol in response to inflammasome agonists is reportedly difficult (***Nature***, 356; 768–74, 1992, ***J. Immunol.***, 159; 5964–72, 1997, ***Cell***, 78; 343–52, 1994). In addition, extracellular mature caspase-1 has been reported to serve as a marker of inflammasome activation *in vitro* (***J. Biol. Chem.***, 274; 36944–36951, 1999). Therefore, we detected immunoprecipitated cleaved caspase-1 p20 in the culture supernatants of *S*. Typhimurium-infected BMDMs using 10% TCA. This is included in the revised Materials and Methods section, as follows:

Line 743: Mature caspase-1 subunits are released into the supernatant in response to inflammasome activation (***J. Biol. Chem.***, 274; 36944-51, 1999); therefore, to detect cleaved caspase-1 p20, cell culture supernatants were precipitated with 10% trichloroacetic acid on ice for 1 h and then centrifuged at 10,000 × *g* at 4°C for 30 min.

***Comment 8.***　 *Expression of SPI-1 genes in Salmonella were previously shown to modulate SPI-1 gene expression (PMID: 19433544, PMID: 12453229), but this is not mentioned in the manuscript nor are these papers referenced. Since the SPI-1 T3SS induces inflammasome activation, this is important to address.*

**(Response)**

Thank you for these important comments. To examine the involvement of SPI-1 in the enhanced inflammasome activation by SCFAs, additional experiments were conducted using *S*. Typhimurium SPI-1 knock-down strains. These results are shown in Supplementary Figure 6 and are discussed as follows:

Low concentrations of SCFAs in streptomycin-treated mice enhanced colonization in the ileum by increasing SPI-1 expression, whereas SCFAs at a concentration of 30 mM decreased the expression of SPI-1 genes *in vitro* (***Infect. Immun.***, 77; 2691-26702, 2009, PMID: 19433544, ***Mol. Microbiol.***, 46; 1451-64, 2002, PMID: 12453229). Our results showed that SCFAs at a concentration of 10 mM enhanced the elimination of *S*. Typhimurium in BMDMs by promoting inflammasome activation through ASC-dependent mechanisms. Therefore, we examined the effect of SCFAs at a concentration of 10 mM on the expression of SPI-1 genes in *S*. Typhimurium strain A. The expression of SPI-1 genes (*sipD* and *prgH*) did not change by treatment with 10 mM propionate, 10 mM butyrate, or 10 mM lactate (S6A Fig), indicating that the enhanced elimination of *S*. Typhimurium in BMDMs by 10 mM SCFAs is independent of the down-regulation of SPI-1 genes *in vitro*. We also evaluated the effect of SCFAs on inflammasome activation in BMDMs using *S*. Typhimurium SPI-1 knock-down strains. The SPI-1 knock-down strain of *S*. Typhimurium was constructed by introducing an in-frame deletion of *sipB*, a transcriptional activator of SPI-1, and we confirmed that the expression of SPI-1 genes (*sipD* and *prgH*) was significantly repressed in *S*. Typhimurium SPI-1 knock-down strains (S6A Fig). When using the SPI-1 knock-down strain of *S*. Typhimurium, the infection number in BMDMs and LDH release were not changed by *ASC* knockout, and only a weak effect by SCFAs was observed in wild-type BMDMs (S6B and S6C Fig). On the other hand, the IL-1β production from wild-type BMDMs, but not *ASC*^−/−^ BMDMs, infected with *S*. Typhimurium SPI-1 knock-down strains, was significantly increased by treatment with 10 mM propionate or 10 mM butyrate (S6D Fig). These results suggest that SCFAs can enhance the production of IL-1β through an ASC-dependent mechanism in macrophages infected with SPI-1 knock-down *S*. Typhimurium, but the pyroptosis associated with the elimination of the *S*. Typhimurium SPI-1 knock-down strain is independent of ASC. These data have been added to the revised version of the manuscript as Supplementary Figure 6, and the Results and Materials and Methods sections have been revised, as follows:

Line 571: **Construction of the *S*. Typhimurium SPI-1 knock-down mutant strain.** SPI-1 knock-down bacteria were constructed with an in-frame deletion of *sipB*, a transcriptional activator of SPI-1. An FRT (Flp recombination target)-flanked chloramphenicol-resistant marker cassette (KD3) fragment was amplified using the primers 5'-GTGTAGGCTGGAGCTGCTTC-3' (forward) and 5'-ATGGGAATTAGCCATGGTCC-3' (reverse) (***J Med Microbiol.***, 64; 295-302, 2015), and the PCR products were inserted into pGFPmut 3.1 (Clonetech, cat. no. 632370) downstream of the *gfp* gene. The *sipB*::*gfp*-KD3 fragment was amplified using the primers 5'-CGGAGACAGAGCAGCACAGTGAACAAGAAAAGGAATAATTATGCGTAAAGGAGAAGAACT-3' (forward) and 5'-TACTAATTAACATATTTTTCTCCCTTTATTTTGGCAGTTTGTGTAGGCTGGAGCTGCTTC (reverse). The PCR products of the *sipB*::*gfp*-KD3 fragment were introduced into *S*. Typhimurium SR-11 χ3306/pKD46 by electroporation to construct the *sipB* in-frame replacement strain of *S*. Typhimurium SR-11 χ3306. The in-frame *sipB* deletion on the chromosome of *sipB*::*gfp*-KD3 fragment-electroporated *S*. Typhimurium SR-11 χ3306 was accomplished by λ Red-mediated recombination (***PNAS***, 97; 6640-6645, 2000).

***Comment 9.*** *Fig S3A: Why are Salmonella killed even in the absence of SCFAs during intracellular residence even at time points where no pyroptosis is detected?*

**(Response)**

This is an important comment. Birmingham *et al*. reported that autophagy activation in response to the damaged *Salmonella*-containing vacuole (SCV) eliminates intracellular *S*. Typhimurium (***J. Biol. Chem.***, 281; 11374-11383, 2006). Therefore, we believe the reason *S*. Typhimurium are killed in the absence of SCFAs during intracellular residence, even at time points where no pyroptosis is detected, is the elimination of intracellular *S*. Typhimurium by autophagy. Further investigation is needed to elucidate the induction mechanisms of autophagy in *S*. Thypimurium infected macrophages.

***Comment 10.*** *Are the mice in Fig 4C and Fig 6 pre-treated with antibiotics?*

**(Response)**

Yes. In Fig. 4C and Fig. 6, to exclude the effects of endogenous SCFAs derived from gut microbiota, antibiotics were administrated for 1 month prior to SCFA administration. To clarify this point, we revised the legends for these figures, as follows:

　　 Line 970: The mice were administrated with antibiotics for 1 month prior to being given drinking water containing 300 mM propionate, 300 mM butyrate, or 300 mM lactate for 1 week. Then, mice were inoculated with *S*. Typhimurium (10^8^ bacteria) orally.

Line 988: Mice were administrated with antibiotics for 1 month prior to being given drinking water containing 300 mM propionate or 300 mM butyrate for 1 week. Then, clodronate liposomes (56 mg per kg of body weight) were intraperitoneally administrated to the mice 24 hours prior to oral infection with *S*. Typhimurium (10^8^ bacteria).

***Comment 11.***  *In Barthel et al 2003 I&I (PMID: 12704158), Salmonella infection in the absence of antibiotic pre-treatment did not lead to cecal inflammation, which is at odds with Fig 5A.*

**(Response)**

Thank you for pointing this out. We have added a discussion of these findings to the revised manuscript:

“Our results show that gut commensal bacteria-derived SCFAs enhance cecal inflammation in S. Typhimurium-infected mice in an ASC-dependent manner. The concentrations of propionate and butyrate in the cecal lumen of the SPF-C57BL/6 mice used in this study were the same as those of antibiotic-treated mice administrated with exogenous SCFAs, which also induced inflammation (S9 Fig). Therefore, the localization of these SCFAs in the cecal lumen of SPF-C57BL/6 mice is thought to induce inflammation in the cecum in response to S. Typhimurium infection (Fig 5A). On the other hand, Barthel et al. reported that S. Typhimurium infection in SPF mice did not lead to cecal inflammation (**Infect. Immun.**, 71; 2839–2858, 2003, PMID: 12704158). SCFAs are metabolites of gut commensal bacteria, and therefore the concentration of SCFAs in the gut depends on the composition of the gut microbiota. Thus, the induction of cecal inflammation in SPF mice infected with S. Typhimurium may depend on the composition of the gut microbiota. The composition of gut commensal bacteria is reported to be significantly different in mice obtained from different vendors (**Comp. Med.**, 60; 336-47, 2010). While further study is needed to clarify the composition profile of gut microbiota associated with the induction of cecal inflammation in response to S. Typhimurium infection, our findings show that SCFAs induce cecal inflammation, which contributes to protection against bacterial infection through an ASC-dependent mechanism.”

***Comment 12.***　*Does SCFA treatment of naïve mice alter tissue architecture?*

**(Response)**

Thank you for this question. To examine whether treatment of SPF mice without *S*. Typhimurium infection with SCFAs alters the tissue architecture, we conducted additional experiments to analyze cecum tissues from SCFA-treated SPF wild-type or *ASC*^−/−^ mice without *S*. Typhimurium infection. Administration of SCFAs to either SPF strain did not induce inflammatory cell infiltration into the cecum mucosa or submucosa, suggesting that SCFAs do not induce cecal inflammation without pathogenic bacterial infection. These data have been added to the revised manuscript as Supplementary Figure 13, and the Results section has also been revised:

Line 326: To determine whether administration of SCFAs to SPF mice without *S*. Typhimurium infection induces inflammatory cell infiltration, cecum tissues from SCFA-treated SPF wild-type or *ASC*^−/−^ mice without *S*. Typhimurium infection were analyzed. Inflammatory cell infiltration was not detected in the cecal mucosa or submucosa of either mouse strain, indicating that SCFAs do not induce cecal inflammation without notable pathogenic bacterial infection (S13 Fig).

***Comment 13.***　*Where is the Salmonella replicating in clodronate-treated mice? Inside other cell types? Extracellularly?*

**(Response)**

Thank you very much for this interesting comment. To assess the localization of *S*. Typhimurium in the cecal tissues of mice administrated with clodronate-encapsulated liposomes, the cecal mucosa was immunostained for *S*. Typhimurium. While colocalization of *S*. Typhimurium and infiltrating macrophages (F4/80-positive cells) was detected in the cecal mucosa of SPF mice that had not been administered clodronate-encapsulated liposomes, increased staining of *S*. Typhimurium in the extracellular space was observed in the cecal submucosa of SPF mice treated with clodronate-encapsulated liposomes (S14 Fig). This result indicates that *S*. Typhimurium can invade and replicate extracellularly in the cecal submucosa of mice without macrophages. This information has been added to the revised manuscript as Supplementary Figure 14.

*Minor comments:*

*1) Needs to be made clear that bacteria do not make SCFAs only to regulate immune responses (pg. 11 lines 62-3).*

**(Response)**

Thank you very much for this comment. This description ["The gastrointestinal microbiota produces short-chain fatty acids (SCFAs) to regulate immune responses"] has been revised as follows: “Short-chain fatty acids (SCFAs) produced by gastrointestinal microbiota regulate immune responses.”

*2) Grammatical errors are found in the manuscript. For example, pg 11 line 64.*

**(Response)**

Thank you very much for comment. This text has been corrected and now reads as follows: "Unbiased screening using SCFA-conjugated affinity nanobeads identified apoptosis-associated speck-like protein (ASC), an adaptor protein of inflammasome complex, as a non-canonical SCFA receptor besides GPRs."

*3) Pg 13 line 107, should be SCFA-bound, not SCFA-found.*

**(Response)**

Thank you very much for comment. This has been corrected.

*4) Fig 4B, "scarify" would be "sacrifice".*

**(Response)**

Thank you very much for comment. This has been corrected.
