# Supplementary material for: Short-chain fatty acids bind to apoptosis-associated speck-like protein to activate inflammasome complex to prevent Salmonella infection
Source: PLoS Biol. 2020 Sep 29;18(9):e3000813. doi: 10.1371/journal.pbio.3000813 (PMC7524008; doi:10.1371/journal.pbio.3000813)
Supplement: Attachment — Submitted filename: PbyP response to the latest comments(PBIOLOGY-D-20-00270R3).docx [file pbio.3000813.s018.docx]

**Reviewer #1:** *The authors very carefully and thoughtfully revised their manuscript. They added a large amount of additional experimental data to support their hypothesis and address the reviewer's concerns. The additional clarifications helped to make the manuscript more accessible to a larger readership. I have only two minor comments.*

**(Response)**

We appreciate the reviewer’s further critical comments and insightful suggestions. We have taken two specific comments from the reviewer into account in the revised manuscript. Detailed responses are provided below.

**Comment 1.** *The authors added a microbiome analysis as requested. Unfortunately, the graphs that were chosen for the manuscript do not actually show that there is no difference in the microbiome composition. The graphs show that there is no difference in alpha diversity, which measures the number and abundance of taxa in a given sample. Alpha diversity can be exactly the same in individuals of two groups while the community composition (which taxa are present) between the groups can be completely different. In order to show that there are no significant differences in community structure between the experimental groups, beta diversity needs to be shown. The microbiota analysis is clearly not the focus of the paper or the expertise of the authors. However, the authors went through the trouble of adding this important piece of data for their mouse experiments. It should therefore be actually significant data that supports their hypothesis. The microbiome data are already available, so adding beta diversity PCA plots should be a minor modification.*

**(Response)**

Thank you very much for this important comment. According to your suggestion, we added a beta diversity principal coordinate analysis (PCoA) plots to Supplementary Figure 8B. The results of PCoA plots showed no difference in the microbiome composition between wild-type and *ASC*^−/−^ mice, suggesting that the gut microbiota has a similar pattern between wild-type and *ASC*^−/−^ mice. This data was shown as Supplementary Figure 8B of the revised manuscript, and the Results and Methods sections have been revised to include these data, as follows:

Line 314: There was no significant difference in the diversity of gut microbiota between wild-type and *ASC*^−/−^ mice in the Shannon diversity index and the Inverse Simpson index results (S8A Fig). Moreover, the results of the principal coordinate analysis (PCoA) plots also showed no difference in the microbiome composition between wild-type and *ASC*^−/−^ mice (S8B Fig). These results suggest that the increased susceptibility of SPF *ASC*^−/−^ mice to *S*. Typhimurium depends directly on ASC, not on a difference in the microflora.

Line 772: The PCoA analysis of the weighted UniFrac distances was applied to the distance matrices for visualization of the microbial diversity of the OTUs in the libraries.

**Comment 2.** *The description of the "boost" is misleading. In the results section, the concentrations of the SCFAs in water are not given, only the concentrations for the "boost". This gives the impression that the concentration given by oral gavage is higher than what the mice are drinking throughout the experiment. Through the clarification in M&M I learned that this is not the fact, that the concentration in the boost and in the water are the same. As you explained in the reply to my comment, this was more a "forced drinking" to prevent differences in measurements resulting from that once mouse had recently drank and another an hour ago. You might want to clarify this in the results section.*

**(Response)**

Thank you for this comment. According to your suggestion, we revised the Results section as follows:

Line 324: Next, the antibiotic-treated mice were given drinking water containing 300 mM SCFAs (propionate or butyrate) or 300 mM lactate for 1 week prior to *S*. Typhimurium infection (Fig. 4B). Treatment with antibiotics significantly reduced the concentrations of propionate and butyrate in the cecal lumen (S9 Fig). It was difficult to determine the exact concentration of the SCFAs of the cecal lumen of the mice given the drinking water containing 300 mM SCFAs because the timing and amount of water intake are different in each mouse, resulting in inconsistent data. Therefore, to measure the concentration of SCFAs in the cecal lumen of the mice immediately after exogenous administration of SCFAs, the mice were forced to drink orally with 100 μL of 300 mM SCFAs 2 h prior to sacrifice, and the SCFA concentrations in the cecal lumen were measured. The exogenous administration of SCFAs restored their concentrations to the levels observed in SPF mice not treated with antibiotics (S9 Fig).

**Reviewer #2**

**Comment 1.** *Why is intracellular growth data presented as % CFU in Fig 3 but as CFU/mL in Fig S6B? Also, line 257 should read "non-significant effect" rather than "weak effect".*

**(Response)**

Thank you for these comments. We revised Fig. S6B to show the data in the same format as in Fig 3, where the CFU is expressed as a percent of the control (% CFU). In addition, we deleted the description "weak effect" to be rephrased as "non-significant effect" in line 290.

**Comment 2.** *The SPI-1 knockdown mutant induced ASC-dependent IL-1β in BMDMs synergistically with SCFAs, but SCFAs did not potentiate LDH release nor diminish intracellular growth of the SPI-1 mutant. This is in contrast to WT bacterial infection during SCFA treatment, where SCFAs led to significantly less intracellular growth of WT Salmonella and more LDH and IL-1β release. This result is not sufficiently explained.*

**(Response)**

Thank you for this comment. In the revised version, we added the explanation of this result to the Discussion section as follows:

Line 594: The SPI-1 of *S*. Typhimurium is known to induce the activation of the inflammasome, and therefore, in macrophages infected with *S*. Typhimurium SPI-1 deletion mutant strains, it has been reported that IL-1β production is small amounts and caspase-1 cleavage is undetectable (***Front Microbiol.***, 2: 8, 2011, PMID: 21687405, ***Nat. Immunol.*** 7, 569–575, 2006, PMID: 16648853). Our results show that SCFAs enhanced LDH release and IL-1β production via an ASC-dependent mechanism in BMDMs infected with *S*. Typhimurium wild-type strain, leading to significantly less intracellular bacterial growth (Fig. 3). On the other hand, SCFAs did not enhance the LDH release and IL-1β production in BMDMs without bacterial infection (Fig. 3). From these results, SCFAs were conceivable to promote the inflammasome activation induced by SPI-1 through mechanisms involving binding to ASC in *S*. Typhimurium-infected macrophages. While SCFAs enhanced IL-1β production in BMDMs infected with SPI-1 knock-down *S*. Typhimurium through an ASC-dependent mechanism, the levels of IL-1β production were lower compared with that of BMDMs infected with *S*. Typhimurium wild-type strains (Fig 3D and S6D Fig). From these findings, it was thought that SCFAs cannot enhance the pyroptosis contributing to the reduction of intracellular bacterial growth in BMDMs infected with *S*. Typhimurium SPI-1 knock-down strains.

***Comment 3.*** "Butyrate" is misspelled in line 143.

**(Response)**

Thank you for this comment. We have corrected the description "Butyrate" in line 114.

***Comment 4.*** Grammatical errors persist.

**(Response)**

Thank you for this comment. We carefully checked the manuscript, and it was proofread by a native speaker.
